# Metabolomics Profiling Reveals Critical Roles of Indoxyl Sulfate in the Regulation of Innate Monocytes in COVID-19

**DOI:** 10.3390/cells14040256

**Published:** 2025-02-11

**Authors:** Liqing He, Yunke Wang, Fang Yuan, Samantha Morrissey, Anne E. Geller, Xiaoling Hu, Raobo Xu, Xipeng Ma, Huang-ge Zhang, Kenneth McLeish, Jiapeng Huang, Xiang Zhang, Jun Yan

**Affiliations:** 1 Department of Chemistry, University of Louisville, Louisville, KY 40292, USA; liqing.he@louisville.edu (L.H.); raobo.xu@louisville.edu (R.X.); xipeng.ma@louisville.edu (X.M.); xiang.zhang@louisville.edu (X.Z.); 2 Immuno-Oncology Program, Brown Cancer Center, Division of Immunotherapy, MD Department of Surgery, University of Louisville, Louisville, KY 40292, USAxiaoling.hu@louisville.edu (X.H.); 3 Department of Microbiology and Immunology, University of Louisville, Louisville, KY 40292, USA; 4 Division of Nephrology and Hypertension, Department of Medicine, University of Louisville, Louisville, KY 40292, USA; kenneth.mcleish@louisville.edu; 5 Department of Anesthesiology and Perioperative Medicine, University of Louisville Hospital, Louisville, KY 40292, USA

**Keywords:** COVID-19, metabolism, indoxyl sulfate, monocytes, immune regulation

## Abstract

The severe acute respiratory syndrome coronavirus 2 (SARS-CoV-2) infection is intricately related to the reprogramming of host metabolism. However, existing studies have mainly focused on peripheral blood samples and barely identified specific metabolites that are critically involved in the pathology of coronavirus disease 2019 (COVID-19). In the current small-scale study, we performed metabolic profiling in plasma (*n* = 61) and paired bronchoalveolar lavage fluid (BALF) samples (*n* = 20) using parallel two-dimensional liquid chromatography–mass spectrometry (2DLC-MS). In addition, we studied how an identified metabolite regulates the immunopathogenesis of COVID-19. The results unveiled distinct metabolome changes between healthy donors, and moderate and severe patients in both plasma and BALF, indicating that locations and disease severity play critical roles in COVID-19 metabolic alteration. Notably, a vital metabolite, indoxyl sulfate, was found to be elevated in both the plasma and BALF of severe COVID-19 patients. Indoxyl sulfate selectively induced TNF-α production, reduced co-stimulatory signals, and enhanced apoptosis in human monocytes. Moreover, its levels negatively correlated with the strength of co-stimulatory signals and antigen presentation capability in monocytes of COVID-19 patients. Collectively, our findings suggest that the levels of indoxyl sulfate could potentially serve as a functional biomarker to monitor COVID-19 disease progression and guide more individualized treatment for COVID-19 patients.

## 1. Introduction

Coronavirus disease 2019 (COVID-19), caused by the severe acute respiratory syndrome coronavirus 2 (SARS-CoV-2) infection, remains an unprecedented threat worldwide [1]. As of 22 December 2024, the World Health Organization (WHO) reported that the disease has claimed more than 7 million lives worldwide. Despite significant progress made in reducing the severity of SARS-CoV-2 infection with vaccines and antiviral therapies, a fundamental gap in knowledge persists regarding the cellular and molecular mechanisms underlying SARS-CoV-2 pathogenesis and immunopathology.

Metabolomics has been utilized for sample profiling with the goal of biomarker discovery in COVID-19. Indeed, many metabolic biomarkers have been identified [2]. Previous studies have found that the levels of α-hydroxyl acids, the catabolic products of amino acids, are increased with disease severity [3]. Phenylalanine, alanine, citrulline, and proline contribute mostly to the metabolic variability in COVID-19 patients [4]. The levels of bile acids, fatty acids, lipids, tryptophan, and arginine are also altered during SARS-CoV-2 infection [5,6,7,8,9]. Furthermore, kynurenine (KYN) has been proposed as a marker for late-stage patients [10], and metabolites in phenylalanine metabolism and purine metabolism pathways, such as phenylalanine, phenylacety-L-glutamine, inosine, and xanthine, have been suggested as early prognosis biomarkers, as their levels change significantly but return to normal at hospital discharge [11]. Coupling metabolomics with other omics studies has helped identify key mediators of pathogenesis between adult and pediatric populations [5]. Interestingly, a machine learning approach has also been recently employed to identify potential top small-molecule metabolites for predicting COVID-19 [12]. However, there are significant challenges in translating these findings into a clinical treatment and/or diagnosis, as metabolomics-identified biomarkers can be substantially impacted by various factors, such as comorbidities [13]. Additionally, most of those studies used serum/plasma samples [14], and their results are correlative. Therefore, it remains unclear whether the metabolites identified from peripheral blood contribute directly to SARS-CoV-2 pathology such as inflammation in the lungs, which can result in acute respiratory distress syndrome (ARDS) in severe COVID-19 patients.

In this study, we collected plasma and bronchoalveolar lavage fluid (BALF) samples to perform metabolic profiling on polar metabolites using parallel two-dimensional liquid chromatography–mass spectrometry (2DLC-MS). Strikingly, we identified one predominate metabolite, indoxyl sulfate (IS), which was elevated in both plasma and BALF from severe COVID-19 patients. Interestingly, IS specifically modulated innate monocyte function to induce cytokine release and apoptosis while it downregulated antigen presentation capability. These findings provide insight into the metabolic mechanism in patients with COVID-19.

## 2. Materials and Methods

### 2.1. Chemicals and Reagents

A total of 363 authentic standards of metabolites were purchased from Fisher Scientific (Loughborough, UK), Sigma-Aldrich Corp. (St. Louis, MO, USA), Cayman Chemical (Ann Arbor, MI, USA), Biosynth International, Inc. (San Diego, CA, USA), and Toronto Research Chemicals Inc. (Toronto, ON, Canada).

### 2.2. Patient Recruitment and Sample Collection

Patient recruitment followed the same criteria in our previous study [15]. Inclusion criteria were hospitalized adults (older than 18) who had positive COVID-19 results and consented to be enrolled in this study. Healthy donors (HD) were healthy adults (older than 18) who had negative COVID-19 results. The diagnosis of COVID-19 was based on a 2019-CoV detection kit using real-time reverse-transcriptase PCR performed at the University of Louisville hospital laboratory from nasal pharyngeal swab samples obtained from patients. The grouping of COVID-19 patients into moderate versus severe groups was based on the initial clinical presentation at the time of enrollment. Moderate group participants were patients with confirmed COVID-19 who were hospitalized without mechanical ventilation. Severe group participants were patients with confirmed COVID-19 who required mechanical ventilation. Peripheral blood and bronchoalveolar lavage fluid (BALF) of COVID-19 patients and control subjects were collected after obtaining written, informed consent. The methods were performed following the approved guidelines from the University of Louisville IRB (#20.0321). The subjects were recruited between April 2020 and December 2020.

### 2.3. Plasma, PBMC, and Monocyte Isolation

Whole blood samples were centrifuged at 541 g for 10 min. Plasma was aspirated and aliquoted into 1 mL Eppendorf tubes and immediately stored at −80 °C until future use. The remaining cell layers were diluted with an equal volume of complete RPMI 1640. The blood suspension was layered over 5 mL of Ficoll-Paque (Cedarlane Labs, Burlington, ON, Canada) in a 15 mL conical tube. Samples were then centrifuged at 845× *g* for 30 min at room temperature without brake. The mononuclear cell layer was transferred to new 15 mL conical tubes and washed with complete RPMI 1640. The cell pellet was resuspended in 3 mL of RPMI 1640 and counted for sample processing. Monocytes were positively separated from peripheral blood mononuclear cells (PBMCs) with anti-CD14 magnetic beads (Miltenyi Biotec, Auburn, CA, USA).

### 2.4. BALF Sample Preparation

Nonbronchoscopic protected BAL was performed using a closed suction system with a 14 F 40 cm catheter inside to prevent aerosolization. After the injection of 30–40 mL sterile normal saline into the endotracheal tube, the suction catheter was inserted through the endotracheal tube and blindly advanced into the distal airways until resistance was felt. The catheter was wedged in that position, and the aspirate was collected in a sterile container into a sputum trap cup. The procedure was repeated if the aspirated fluid was less than 5 mL.

### 2.5. Metabolite Extraction

Polar metabolite extraction was performed using the Bligh and Dyer method [16] with minor modifications. Specifically, plasma samples were diluted 2.67 times with water. Four hundred μL of diluted plasma or BALF samples was mixed with 1.5 mL chloroform–MeOH (1:2, *v*/*v*) with 1 mM butylated hydroxytoluene (BHT). The mixture was vigorously vortexed for 1 min and then 0.5 mL chloroform was added. After vortexing for 1 min, 0.5 mL water was added and vortexed for another 1 min. After centrifugation at 1300 g for 8 min, 0.8 mL of the top layer from plasma and 0.85 mL of the top layer from BALF samples were collected and dried by speed vacuum at 4 °C followed by lyophilization. The dried sample was re-dissolved in 200 µL (for plasma) or 100 µL (for BALF) 50% acetonitrile for 2DLC-MS analysis.

### 2.6. DLC-MS/MS Analysis

2DLC-MS and 2DLC-MS/MS analyses were performed using a parallel method as described previously [17]. The group-based pooled sample was analyzed by 2DLC-MS/MS to acquire MS/MS spectra at four collision energies (10, 20, 40, and 60 eV) for metabolite identification. A pooled sample was also analyzed by 2DLC-MS after analyzing every six biological samples for quality control.

### 2.7. Indoxyl Sulfate (IS) and TNF-α Quantification by ELISA

Quantification of IS (MyBioSource, San Diego, CA, USA) in human plasma and TNF-α (BioLegend, San Diego, CA, USA) production in monocytes were analyzed using ELISA kits. The operating procedure provided by the manufacturer was followed. Purified monocytes were stimulated with IS at indicated concentrations for 3 days. Fifty μL of plasma or 100 μL of supernatant was used for each sample. The OD at 450 nm was measured using a Synergy HT microplate reader (BioTek, Layton, UT, USA). Concentrations were determined using the standard curves.

### 2.8. Flow Cytometry

Fluorescent antibodies were purchased from Biolegend (CD14, TNF-α, CD3, CD4, CD8, CD56, CD19, HLA-DR, CD80, CD86, CD33, CD40). For cell surface marker staining, cells were first treated with Fc Blocker (Biolegend) and then stained with cell surface antibodies at 4 °C for 20 min. The relevant isotype control antibodies were also used. For TNF-α staining, cells were cultured with IS for 2 days. Golgi-plug was added for the last 5 h. Cells were stained with surface antibodies followed by fixation, permeabilization (Biolegend), and intracellular staining of TNF-α at 4 °C overnight. Data were acquired by a FACSCanto (BD Biosciences, Franklin Lakes, NJ, USA).

### 2.9. t-SNE Analysis of Monocytes Clusters

Upon acquisition of the samples by flow cytometry, the fcs files were analyzed using FlowJo10 and the FlowSOM. t-SNE plots were generated using FlowJo software 10.10.

### 2.10. Annexin V/7AAD Assay

Purified monocytes were stimulated with a vehicle and IS 1 mM for 2 days. Cell apoptosis was detected with Annexin V/7AAD staining according to the manufacturer’s instructions (BD Biosciences). The stained cells were analyzed with flow cytometry.

### 2.11. Human Apoptosis Array

Purified monocytes were stimulated with a vehicle and IS 1 mM for 2 days. Cells were lysed and 100–150 μg protein lysates were used to evaluate the apoptosis-related proteins with a human apoptosis array kit (R&D systems, Minneapolis, MN, USA) strictly according to the manufacturer’s protocol. Membranes were exposed to X-ray film for 10 s to 1 min, and the intensity of proteins was quantified by Image J software (J2).

### 2.12. Data Analysis

XCMS software was used for 2DLC-MS data deconvolution [18]. Compound Discover (version 3.2, Thermo Fisher Scientific, Inc., Bremen, Germany) and MetSign software were used for metabolite identification [17,19]. Statistical analyses were performed using SPSS software (version 25, IBM Corporation, Armonk, NY, USA) and R (version 4.1.0). Distributional assumptions of continuous outcomes were checked, and, if needed, a data transformation (e.g., log transformation) was applied to meet the normality assumption. The data normality test was achieved using the Shapiro–Wilk method. The metabolic profile differences among groups was evaluated using partial least squares-discriminant analysis (PLS-DA). Quantitative pathway enrichment analysis was performed using Metaboanalyst (version 5.0, https://genap.metaboanalyst.ca/MetaboAnalyst/ (accessed on 24 February 2023). Univariate analysis of metabolite abundance among groups was conducted using one-way ANOVA with Tukey post-test and the Benjamini and Hochberg method [20] for multiple testing correction. ROC analysis was employed to evaluate the diagnostic potential of metabolite abundance variations in COVID-19 patients. Spearman’s rank correlation was applied to assess the association of specific compounds between paired plasma and BALF samples and significantly changed metabolites with clinical tests. The thresholds of statistical significance tests were set as *q* ≤ 0.05, and areas under the ROC curve (AUC) > 0.7 or <0.3. The error bars in each histogram plot are the standard error of the mean (SEM).

## 3. Results

### 3.1. Patients, Data Collection, and Study Design

**Figure 1:**
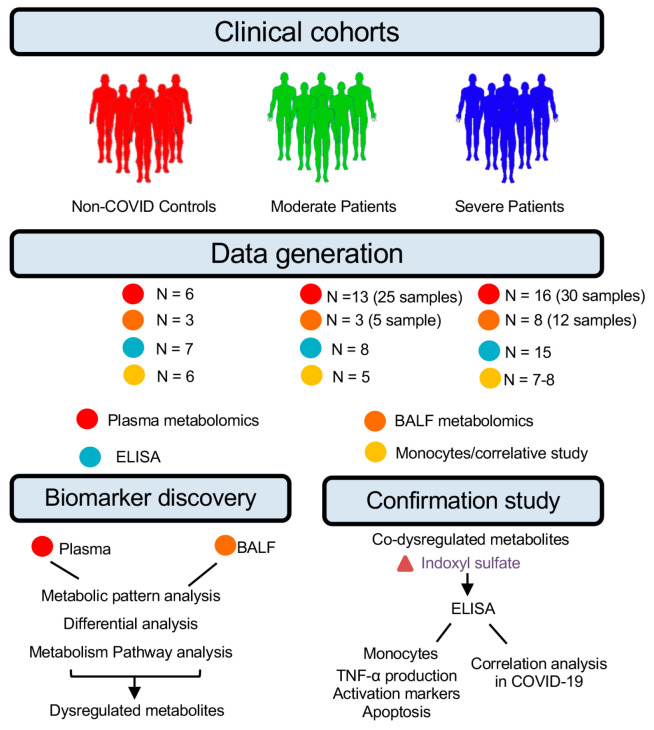
Framework for biomarker discovery and underlying mechanism study in COVID-19. High-throughput metabolomics was used to profile paired BALF and plasma samples from 6 healthy donors, 3 control patients, and 55 COVID-19 patients with different disease severity of infection. Metabolome dysregulation in BALF and plasma was then integrated to capture disease-stage-relevant metabolite signatures in the bloodstream that were concordant with the BALF. The metabolites with significant abundance alteration in both the plasma and BALF were confirmed by ELISA and the mechanism of these changes was studied in the same cohort.

The study population consisted of 29 COVID-19 patients who tested positive for SARS-CoV-2 by nasopharyngeal swab and six healthy donors. We categorized all samples into three groups for the metabolic profiling study: healthy donors (HD, *n* = 6), moderate patients (MP, without mechanical ventilation, *n* = 13), and severe patients (SP, with mechanical ventilation, *n* = 16). Three patients without lung diseases were also recruited to collect BALF as controls (Control) for BALF metabolomics analysis. Some of these patients had 2–3 time-point samples, resulting in a total of 61 plasma samples and 20 BALF samples (Figure 1). Human subjects’ demographic data are summarized in Table 1.

Figure 1 depicts the study design. Untargeted metabolic profiling was carried out on all plasma (*n* = 61) and BALF (*n* = 20) samples. The changes in co-dysregulated metabolites in these two types of samples were confirmed by ELISA. The underlying mechanism of these metabolites was studied in the same cohort of patients.

### 3.2. SARS-CoV-2 Infection Impacts on Plasma Metabolome

**Figure 2:**
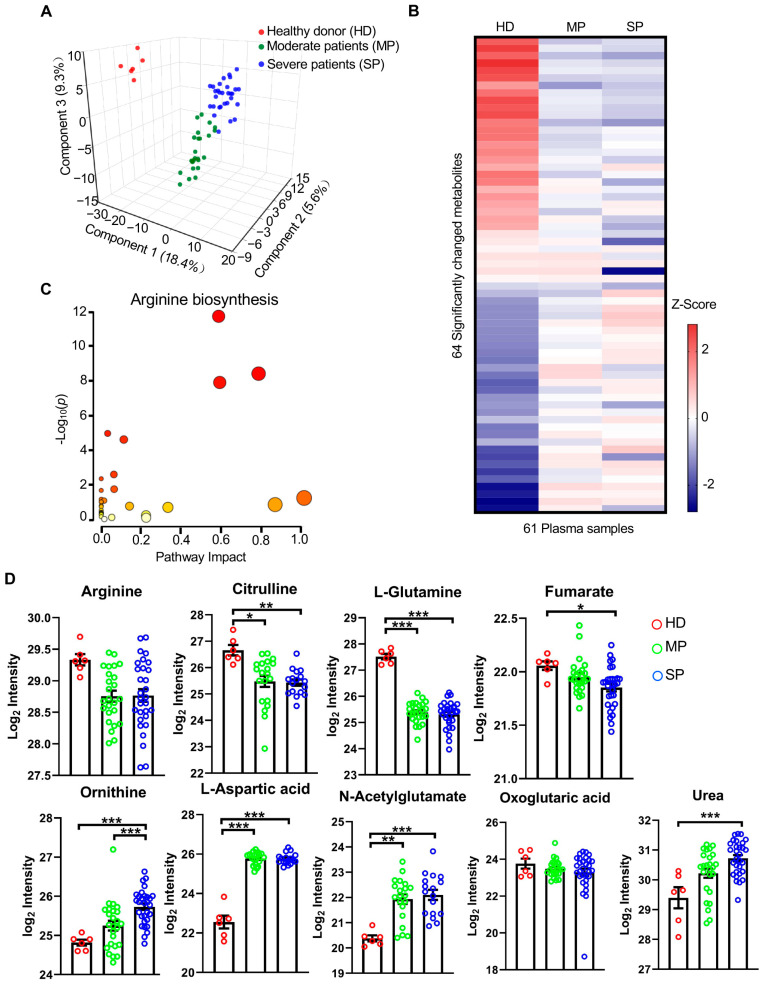
Plasma metabolome remodeling due to disease severity of COVID-19 infection. (**A**) Three-dimensional score plot of PLS-DA analysis using all metabolites detected in plasma by 2DLC-MS under positive and negative modes. (**B**) Heatmap of the 93 significantly dysregulated metabolites in the 61 plasma samples. Row shows median log_2_ peak area after z-score normalization across the three stages. HD, healthy donor; MP, moderate patients; SP, severe patients. (**C**) Pathway analysis using all significantly changed metabolites in plasma. Arginine biosynthesis is the most affected metabolic pathway. The red color indicates the pathway is significantly changed while the size of circle denotes impact on disease development. (**D**) Abundance of metabolites in the arginine biosynthesis pathway. One-way ANOVA was used for statistical significance test with Tukey method for post-hoc analysis and Benjamini and Hochberg method for multiple test correction: * *q* < 0.05; ** *q* < 0.01; *** *p* < 0.001.

A total of 144 metabolites were identified in plasma samples, of which 55 were identified using our in-house database by parent ion *m*/*z*, retention time, and MS/MS spectrum matching, and 89 metabolites were identified using the public database by parent ion *m*/*z* and MS/MS spectrum matching (Table S1). PLS-DA was performed to study the metabolic profile difference among the three groups using the abundance of the identified metabolites. The metabolic profiles were greatly different among the three groups (Figure 2A), with 83 metabolites having a variable importance projection (VIP) scoring greater than 1.0 (Figure 2B). The large values of R^2^ = 0.88 and Q^2^ = 0.64 indicated very good discrimination and predictability of the PLS-DA model.

Differential abundance analysis in the plasma metabolome revealed 51 metabolites significantly dysregulated (*q* < 0.05 and fold-change (FC) > 20%) between patients with different severities (MP and SP) and HD, 9 between SP and HD, and 4 between MP and SP, totaling 64 metabolites accounting for about 45% of the quantified plasma metabolome (Table S2). Among these significantly changed metabolites, 34 of them were increased and 29 metabolites were decreased in patients (MP and SP) (Figure 2B). Pathway analysis showed that several pathways showed highly significant enrichment of metabolites and the arginine biosynthesis pathway is the most affected pathway by SARS-CoV-2 infection (Figure 2C). Four metabolites including arginine, citrulline, L-glutamine, and fumarate in the arginine biosynthesis pathway were significantly decreased (Table S1), while four metabolites including ornithine, L-aspartic acid, N-acetylglutamate, and urea were significantly increased in the patient plasma (Figure 2D).

**Figure 3:**
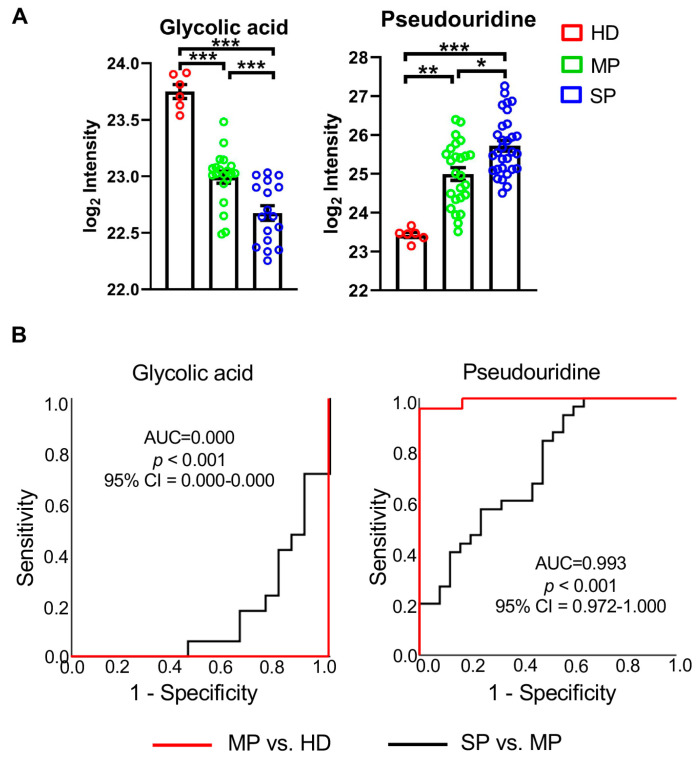
Metabolic biomarkers in plasma for potential diagnosis of severe COVID-19. (**A**) The abundance change of three potential biomarkers in plasma detected by untargeted metabolomics using 2DLC-MS. (**B**) ROC-AUC of the three potential biomarkers. The black curve shows the ROC-AUC of severe patients (SP) vs. moderate patients (MP); the AUC of glycolic acid is 0.156, *p* < 0.001 and 95% CI = 0.031–0.281. AUC of 2-furoic acid is 0.257, *p* = 0.002, and 95% CI = 0.119–0.396. AUC of pseudouridine is 0.727, *p* = 0.004, and 95% CI = 0.592–0.861. The red curve shows the ROC-AUC of MP vs. healthy donors (HD); the AUC of glycolic acid and 2-furoic acid is 0.000, *p* < 0.001, and 95% CI = 0.000–0.000. AUC of pseudouridine is 0.993, *p* < 0.001, and 95% CI = 0.972–1.000. One-way ANOVA was used for statistical significance test with Tukey method for the post-hoc analysis and Benjamini and Hochberg for multiple test correction: * *q* < 0.05; ** *q* < 0.01; *** *q* < 0.001.

Among the 64 differentially expressed metabolites, the abundance levels of two metabolites, glycolic acid and pseudouridine, were altered stepwise with the increase in disease severity (Figure 3). The peak area of glycolic acid was decreased from HD to MP, and ultimately reached its lowest value in the plasma of SP, while the peak area of pseudouridine was increased from HD to MP and reached its highest level in the plasma of SP (Figure 3A). Receiver operating characteristic (ROC) curve analysis was then employed to evaluate the diagnostic performance of these two metabolites, and the result showed that these two metabolites could differentiate SP from MP and MP from HD. Glycolic acid had a prime AUC (AUC = 0.000; 95% CI, 0.000–0.000) in the ROC analysis of MP vs. HD. It also had an excellent AUC (AUC = 0.156; 95% CI, 0.031–0.281) in the ROC analysis of SP vs. MP (Figure 3B). ROC analysis also showed significant abundance changes of pseudouridine in SP when compared to MP with AUC = 0.993 (Figure 3B), suggesting that these two metabolites are closely related to SARS-CoV-2 infection and disease severity.

### 3.3. BALF Metabolome in COVID-19 Patients

To investigate the lung metabolites associated with SARS-CoV-2 infection, BALF samples were collected from three MP and eight SP. Some BALF samples were collected at different time points resulting in a total of 17 samples (Figure 1). In addition, three BALF samples were collected from inpatients who had no evidence of lung disease; therefore, these samples were used as controls (Table 1). BALF metabolites were also quantified by parallel 2DLC-MS. In BALF, a total of 147 metabolites were identified in plasma samples (Table S2). Among all these metabolites, 59 were matched with our in-house database by parent ion *m*/*z*, retention time, and MS/MS spectrum matching, and 88 metabolites were identified from the public database by parent ion *m*/*z* and MS/MS spectrum matching. The three groups showed distinct metabolic profiles with R^2^ = 0.96 and Q^2^ = 0.41 in the PLS-DA model (Figure S1A). Notably, two metabolites, indoxyl sulfate and 4-aminophenol, were significantly changed between groups. The levels of indoxyl sulfate were substantially increased in SP when compared with controls and MP, and the increase was statistically significant in SP vs. MP (Figure S1B), while 4-aminophenol levels were significantly decreased in the BALF of SP vs. controls (Figure S1B).

### 3.4. Integration of Plasma and BALF Metabolomics Reveals Common Metabolites in Both Samples

**Figure 4:**
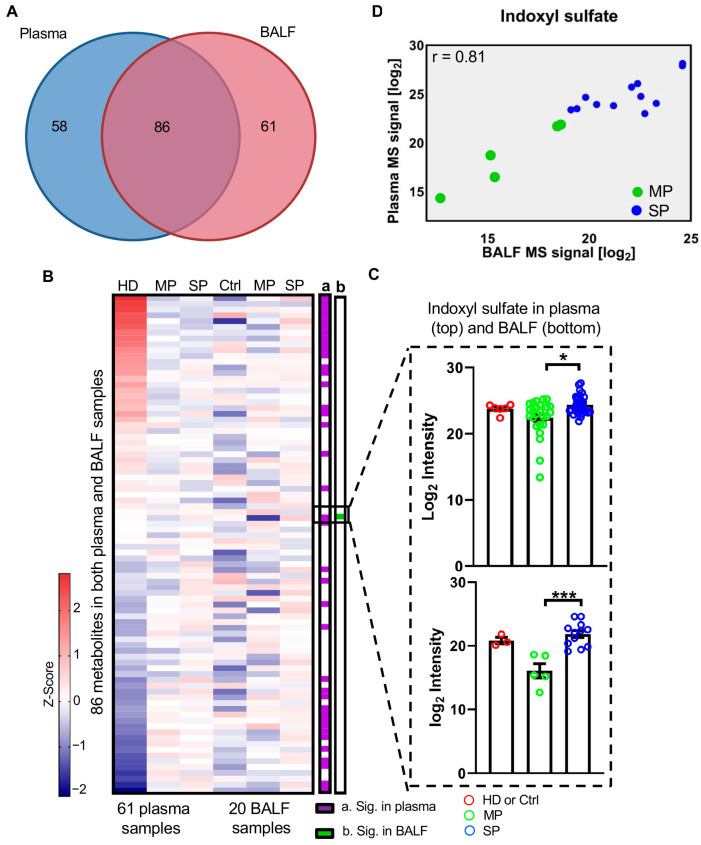
Integration of BALF and plasma metabolomics analyses. (**A**) Overlapping metabolites between BALF and plasma. (**B**) Abundance of all metabolites detected in both BALF and plasma. Row shows median log_2_ peak area of each detected metabolite after z-score normalization across different stages. HD, healthy donor; Ctrl, control patients; MP, moderate patients; SP, severe patients. (**C**) Abundance changes of co-dysregulated metabolites in BALF and plasma. (**D**) Indoxyl sulfate is significantly correlated in paired BALF and plasma samples, with Spearman’s rank correlation coefficient r. One-way ANOVA was used for statistical significance test with Tukey method for post-hoc analysis and Benjamini and Hochberg method for multiple test correction: * *q* < 0.05; *** *q* < 0.001.

We next integrated two metabolomics datasets and found that 86 metabolites were commonly quantified in both plasma and BALF samples (Figure 4A), of which 42 were significantly changed in plasma. However, only one metabolite, indoxyl sulfate, was significantly changed in BALF which is the only metabolite that was dysregulated in both plasma and BALF (Figure 4B). Even though the abundance changes of indoxyl sulfate were not statistically significant in patients (MP or SP) vs. HD, it was notably increased in SP compared to MP with *q* = 0.049 in plasma (Figure 4C, top) and *q* < 0.001 in BALF (Figure 4C, bottom). Figure 3A indicates that indoxyl sulfate has a significant correlation between paired BALF and plasma samples with a Spearman’s rank correlation coefficient r = 0.81.

Metabolites glycolic acid and pseudouridine in plasma could differentiate COVID-19 patients with different disease severities as shown in Figure 3. Pseudouridine was also detected in BALF samples. Pseudouridine in BALF was increased stepwise with disease severity (Figure S1C). Spearman’s rank correlation analysis shows that pseudouridine has a significant correlation (r = 0.62) between paired BALF and plasma samples (Figure S1D). These data collectively suggest that systemic metabolites induced by SARS-CoV-2 infection may also impact local lung metabolic reprogramming.

### 3.5. Indoxyl Sulfate Stimulates TNF-α Production, Downregulates Co-Stimulatory Signals, and Induces Apoptosis in Human Monocytes

**Figure 5:**
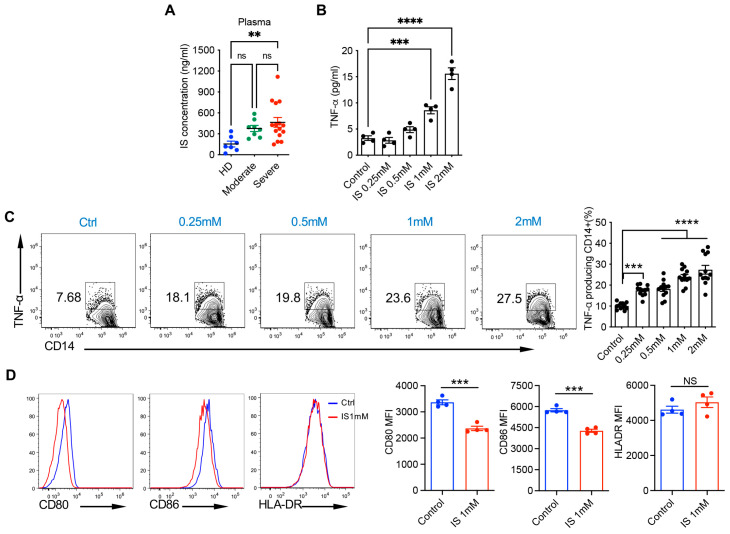
Indoxyl sulfate induces the TNF-α production and weakens co-stimulatory signals in human monocytes. (**A**) Plasma levels of indoxyl sulfate (IS) from healthy donors (HDs, *n* = 7), and moderate (*n* = 8) and severe (*n* = 15) COVID-19 patients were quantified with human IS ELISA. (**B**) Purified monocytes from one HD were stimulated with IS at indicated concentrations for 3 days. TNF-α levels in the supernatant were quantified with ELISA. (**C**,**D**) PBMCs from one HD were stimulated with IS at indicated concentrations for 2 days. Golgi-plug was added for the last 5 h. Representative dot plots of intracellular TNF-α (**C**), and histograms of CD80, CD86, and HLA-DR (**D**) with accompanying summarized data are shown. Flow plots were gated on live CD14^+^ cells. TNF-α ELISA and flow plots are representative of at least three independent experiments with similar results. Data are shown as mean ± SEM. ** *p* < 0.01, *** *p* < 0.001, **** *p* < 0.0001; NS, not significant (one-way ANOVA, unpaired Student’s *t* test).

Since indoxyl sulfate is the sole metabolite that is dysregulated in both plasma and BALF samples, its plasma levels in HD, MP, and SP were further validated using ELISA (Figure 5A). Increased indoxyl sulfate levels were observed in the plasma samples from MP and SP compared to HD, although there was no statistically significant difference between HD and MP. Consistent with previous studies [21,22], we confirmed that indoxyl sulfate could dose-dependently induce TNF-α production in human monocytes, as evidenced by ELISA (Figure 5B) and flow cytometry analysis (Figure 5C). We next investigated the impact of indoxyl sulfate treatment on the expression of HLA-DR and co-stimulatory molecules CD80 and CD86 on monocytes. Following indoxyl sulfate treatment, CD80 and CD86 expression levels exhibited a substantial decrease, while HLA-DR remained relatively unchanged (Figure 5D), indicating that indoxyl sulfate could impair the activation signals of monocytes for downstream T cells.

**Figure 6:**
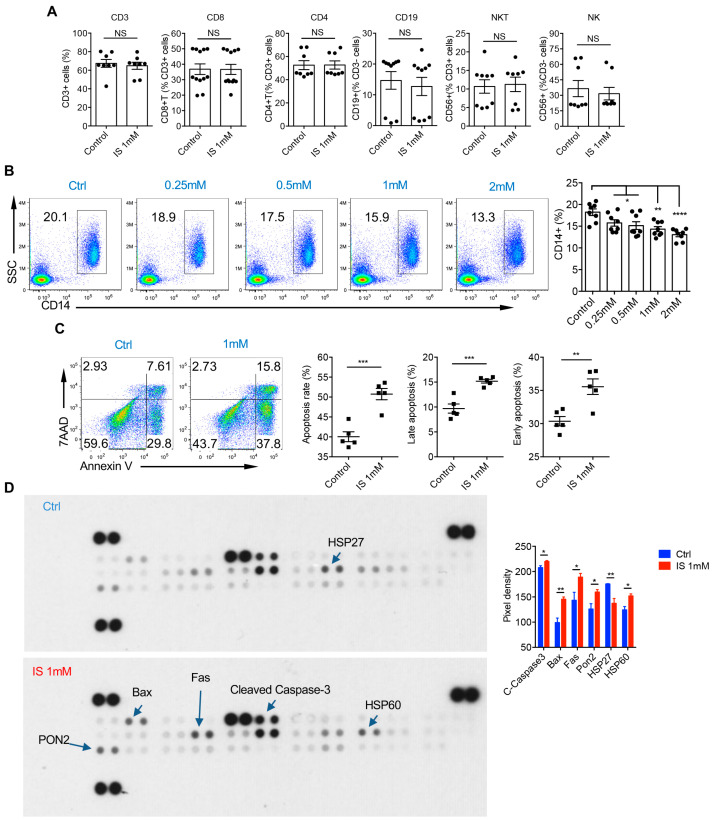
Indoxyl sulfate induces the apoptosis of human monocytes. (**A**,**B**) PBMCs from HDs were stimulated with IS at indicated concentrations for 2 days. (**A**) Summarized percentages of total CD3 cells, CD4 T cells, CD8 T cells, B cells, NKT cells, and NK cells in control and IS 1 mM group (*n* = 8–12). (**B**) Representative dot plots of CD14 with accompanying summarized data are shown. Flow plots are representative of six independent experiments with similar results. (**C**,**D**) Purified monocytes from HDs were stimulated with vehicle and IS 1 mM for 2 days. (**C**) Representative dot plots of late (Annexin V+7AAD+) and early (Annexin V+7AAD-) apoptosis rates using Annexin V/7AAD staining with accompanying summarized data are shown (*n* = 5). Flow plots are representative of three independent experiments with similar results. (**D**) Analysis of apoptosis-related proteins in vehicle and IS 1 mM group using human apoptosis array (*n* = 4). Data are shown as mean ± SEM. * *p* < 0.05, ** *p* < 0.01, *** *p* < 0.001, **** *p* < 0.0001; NS, not significant (unpaired Student’s *t* test).

To investigate whether indoxyl sulfate has any effect on other immune cells, we treated peripheral mononuclear cells (PBMCs) from healthy donor peripheral blood with varying amounts of indoxyl sulfate. The percentages of other immune cell types, such as CD8 and CD4 T cells, B cells, NKT cells, and NK cells, remained unaltered (Figure 6A). However, the percentage of CD14^+^ monocytes decreased in a dose-dependent manner following indoxyl sulfate treatment (Figure 6B). To delve deeper into the effects of indoxyl sulfate on monocytes, we investigated whether it induced apoptosis in these cells. Annexin V/7AAD staining revealed a significant increase in both early and late apoptosis rates in indoxyl sulfate-treated monocytes (Figure 6C). To elucidate the underlying mechanisms of apoptosis induction, we analyzed apoptosis-related proteins using a human apoptosis array. Indoxyl sulfate-treated monocytes exhibited higher expression levels of pro-apoptotic proteins, including cleaved caspase 3, Bax, and the apoptosis inducer Fas (Figure 6D). Furthermore, our findings indicated altered expression of heat shock proteins (HSPs) known to play critical roles in apoptosis regulation. Indoxyl sulfate-treated monocytes displayed reduced HSP27 expression, which has been shown to inhibit the intrinsic apoptotic pathway through interactions with cytochrome c and caspase 3 [23,24], as well as increased HSP60 expression (Figure 6D). HSP60 can exert both pro-apoptotic roles by promoting caspase-3 activation and anti-apoptotic roles by inhibiting Bax activity [25,26]. Surprisingly, the expression of anti-apoptotic protein Pon2 was unexpectedly increased in the indoxyl sulfate-treated group (Figure 6D), possibly indicating a compensatory effect in apoptotic cells. Notably, the control group always exhibited high levels of apoptosis, which was likely attributed to the short survival of ex vivo-cultured human monocytes without any stimulation. These results suggest that indoxyl sulfate can functionally induce TNF-α production in human monocytes and selectively promote their apoptosis.

### 3.6. Indoxyl Sulfate Levels Correlate with the Activation Levels of Human Monocytes in COVID-19 Patients

**Figure 7:**
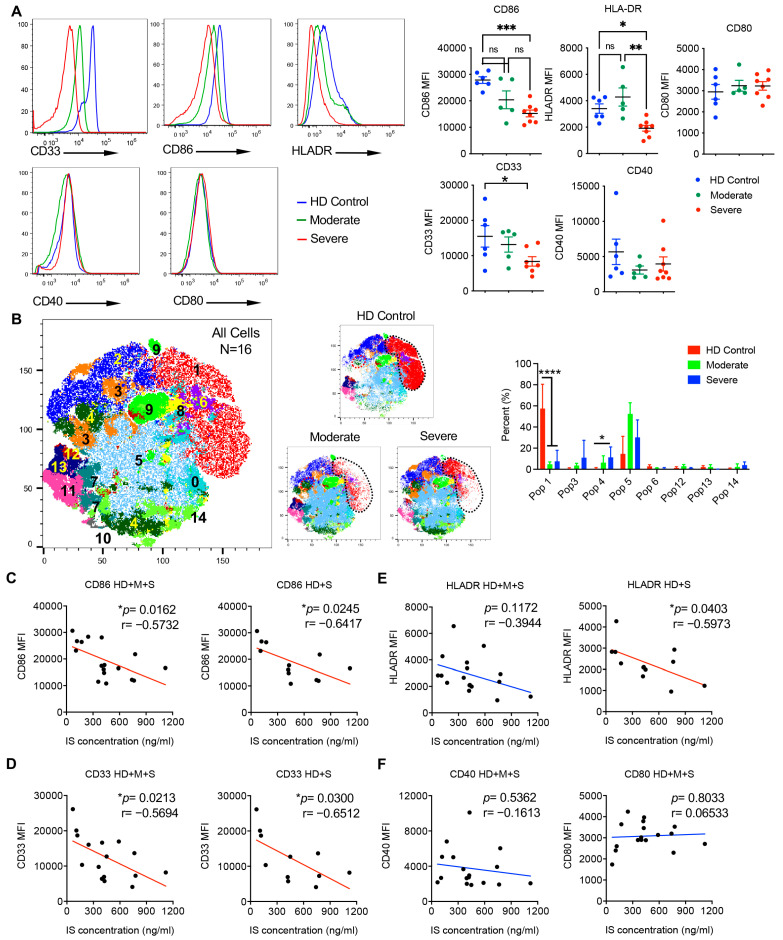
Indoxyl sulfate concentration in plasma correlates with activation marker expressions in peripheral monocytes of COVID-19 patients. (**A**) The expression levels of activation markers CD33, CD86, HLA-DR, CD40, and CD80 in CD14^+^ monocytes from PBMCs of HDs (*n* = 6), and moderate (*n* = 5) and severe (*n* = 7–8) COVID-19 patients. Representative histograms and summarized data are shown. Data are shown as mean ± SEM. * *p* < 0.05, ** *p* < 0.01, *** *p* < 0.001; ns, not significant (one-way ANOVA). (**B**) t-SNE analysis of monocytes in HD (*n* = 6), and moderate (*n* = 5) and severe groups (*n* = 6). t-SNE plot for all cells and representative t-SNE plots from each group (left) and summarized percentage of clusters are shown (right). * *p* < 0.05, **** *p* < 0.0001 (unpaired Student’s *t* test). (**C–F**) The expression levels of indicated activation markers on monocytes were correlated with IS concentrations of the plasma measured by ELISA in the corresponding samples. For all correlation data, a line of best fit is shown to visually depict correlation, with a red line representing a statistically significant correlation and a blue line representing a non-significant correlation. Pearson correlations were used to determine statistical significance in all correlations, where * *p* < 0.05.

We previously observed that indoxyl sulfate treatment could reduce the expression levels of co-stimulatory molecules CD80 and CD86 in monocytes from healthy donors. To explore whether the monocytes from COVID-19 patients exhibit a similar phenotype, we evaluated the expression levels of several activation markers, including CD33, CD86, CD80, HLA-DR, and CD40, in monocytes isolated from the peripheral blood mononuclear cells (PBMCs) of HD, MP and SP group. Remarkably, monocytes from SP patients displayed substantially lower expression levels of CD86, CD33, and HLA-DR compared with those from HD (Figure 7A), similar to indoxyl sulfate-treated monocytes from healthy donors. MP showed no difference with HD in these markers but had higher expression of HLA-DR compared to SP (Figure 7A). CD80 and CD40 expression showed no difference among the three groups. These data reveal the weakened level of antigen presentation and co-stimulation in the monocytes of severe COVID-19 patients. To further explore monocyte heterogeneity, we employed t-SNE analysis (Figure S2A). Different populations were determined by combinatorial marker expression (Figure S2B). t-SNE cluster analysis revealed that the percentage of CD86^+^CD14^+^ monocytes (pop 1) was substantially decreased in MP and SP patients as compared to HD (Figure 7B). In contrast, HLA-DR^low^CD14^+^ monocytes (pop 4) were proportionally increased in SP (Figure 7B).

To assess whether indoxyl sulfate concentrations in plasma could serve as indicators of the activation level of monocytes in COVID-19 patients, indoxyl sulfate concentrations were correlated with the expression levels of activation markers on monocytes from all recruited subjects. The expression levels of CD86 and CD33 were negatively correlated with indoxyl sulfate concentrations in HD + MP + SP and HD + SP (Figure 7C,D). HLA-DR was negatively correlated with indoxyl sulfate concentrations in HD + SP but not in all subjects (Figure 7E). No significant correlations were found between indoxyl sulfate concentrations and CD40 or CD80 expression levels (Figure 7F). We also analyzed the correlations between the abundance of indoxyl sulfate and other significantly altered metabolites with the levels of D-dimer, ferritin, C-reactive protein (CRP), and cytokines including IL-6 in plasma (Table S3). No significant correlations were observed. These findings indicate that the elevated indoxyl sulfate levels might specifically contribute to the impaired activation of monocytes in COVID-19 patients.

## 4. Discussion

In the current study, we employed parallel 2DLC-MS for untargeted metabolomics to achieve the simultaneous separation of metabolites on two orthogonal columns, HILIC and RPC, in one MS analysis. Compared to conventional LC-MS/MS-based untargeted metabolomics [27], this configuration offers several noteworthy advantages. Firstly, parallel 2DLC-MS increases metabolite coverage by allowing the detection of metabolites that may not be retained well in one column but can be effectively separated in the other. Secondly, the simultaneous separation of metabolites on two different columns in a single injection reduces mass spectrometry detection variations and increases the analysis efficiency, contributing to more robust and reliable results. Lastly, the abundance of the same metabolite detected at different retention times can be used to cross-validate the quantification results of that metabolite, further enhancing the confidence in the data obtained. The abundance of metabolites in the samples collected from the same patients at different times was similar suggesting the great robustness and high accuracy of the 2DLC-MS platform. In addition, these data suggest that metabolic changes mediated by SARS-CoV-2 infection are not short-lived and may last a period of time during disease progression.

We used parallel 2DLC-MS to investigate plasma and BALF metabolome changes associated with SARS-CoV-2 infection and identified circulating metabolites with diagnostic value in the detection of the severity of COVID-19. Metabolic profile analysis indicates that metabolites in plasma could separate patients very well by the PLS-DA model, suggesting that the metabolome is very different in the three groups. In addition, almost half of the plasma metabolites quantified in this study are changed, indicating that SARS-CoV-2 infection greatly alters patients’ metabolism. Some of the 64 significantly changed metabolites, such as urea, inosine, xanthine, and alpha-N-phenylacetyl-L-glutamine, were also reported in recent publications [11,28].

Arginine, a node for immune response regulation, serves as a substrate for distinct metabolic pathways that profoundly affect immune cell biology and function [29]. The availability, synthesis, and catabolism of arginine are highly related to immune responses, and its fine-tuning can dictate divergent pro-inflammatory or anti-inflammatory immune outcomes. The host immune response to SARS-CoV-2 plays a critical role in disease pathogenesis and clinical manifestations [30]; therefore, it is reasonable that the arginine biosynthesis pathway is the most affected in patients with COVID-19 (Figure 2C and Figure S1E). Decreased arginine and increased urea in plasma (Figure 2D) and BALF (Figure S1F) indicate increased arginine catabolism in patients. In addition, deprivation of arginine greatly inhibits effector T cell function [31,32], and significantly decreased arginine in both peripheral blood and BALF in severe COVID-19 patients may be directly related to an impaired T cell function as reported previously [33,34,35].

The markers discovered in this study were selected in an unbiased manner, driven by their abundance alteration in groups. The significant alteration between groups and large AUCs of these metabolites indicate their strong ability as a diagnosis method for predicting SARS-CoV-2 infection or determining the severity of patients. Although many assays, such as the rapid PCR assay, have been used for SARS-CoV-2 detection, identifying functional biomarkers related to disease progression and immune status is of great value. The biomarkers’ detection of the different stages of COVID-19 identified in this study have considerably better, or at least comparable, performance compared with existing criterion, especially glycolic acid, which differentiates the severe from the moderate patients according to ROC–AUC analysis.

Integration of BALF and plasma metabolomics results mainly in the identification of upregulated indoxyl sulfate in severe COVID-19 patients. A high Spearman’s correlation coefficient of indoxyl sulfate between plasma and BALF indicates that changes in indoxyl sulfate may be specific in the lungs, not other affected organs, such as the heart or brain. Indoxyl sulfate is produced from the sulfonation of bacterially derived indole by sulfotransferases in the liver [36]. It has been shown to induce immune dysfunction in vascular endothelial cells and immune cells including CD4^+^ T cells and monocytes [21]. Interestingly, indoxyl sulfate is a metabolite derived from L-tryptophan amino acid fermentation [37] and there is a relay pathway between arginine and tryptophan metabolism [38]. It is possible that a decreased arginine level is directly associated with increased indoxyl sulfate in severe COVID-19 patients. As a uremic toxin, indoxyl sulfate is highly expressed in the serum of end-stage renal disease (ESRD) patients [39] and has been reported to cause vascular damage through the TNF-α/CX3CL1 axis [21]. Considering that severe COVID-19 is widely reported to cause acute kidney injury [40,41] and thrombotic events [42,43], it is plausible that indoxyl sulfate is linked to these complications. Furthermore, indoxyl sulfate can induce TNF-α production and enhance apoptosis in human monocytes. Indeed, the combination of TNF-α and IFN-γ has been shown to play a critical role in tissue damage and inflammatory cell death upon SARS-CoV-2 infection via the JAK/STAT1/IRF1 axis [44]. While not as abundant, indoxyl sulfate-induced TNF-α production and cell apoptosis may constitute an important part of the intricate immune network that leads to cytokine storm in severe COVID-19 cases. Moreover, COVID-19 is characterized by pronounced changes in innate immune cells, including aberrant monocytes [45]. In contrast, lymphopenia is a cardinal feature in hospitalized, severe COVID-19 patients [15,46]. Consistent with our findings, previous studies have reported reduced expressions of HLA-DR in circulating monocytes of COVID-19 patients [47,48]. We also observed reduced CD80 and CD86 expression upon indoxyl sulfate treatment, indicating dysregulated functions of antigen presentation and effector T lymphocyte activation in monocytes. The significantly negative correlation between indoxyl sulfate concentrations and HLA-DR, CD86, and CD33 underlines the potential of this metabolite as an immunomodulator and a valuable predictor for monitoring disease severity. It is plausible that indoxyl sulfate levels could serve as a functional biomarker for monitoring disease progression and guiding more individualized treatment for patients with SARS-CoV-2.

There are some limitations in this study. The number of patients included in this study was small, and the control cohort was younger compared to the COVID-19 patients, although the age range for moderate and severe COVID-19 patients was similar (Table 1). A larger cohort with more diverse controls is needed to further validate and strengthen the conclusions of this study. Additionally, many patients had comorbidities such as hypertension and diabetes and comorbidities are also associated with metabolic changes [13]. Given the difficulty of acquiring BALF samples from moderate COVID-19 patients, it will be important to expand these analyses to include more severe COVID-19 patients and non-COVID ARDS patients to ensure that the study’s conclusions are not affected by these confounding factors. In addition, the samples in this study were obtained from patients infected early in the pandemic and medication information is not fully available. It will be of interest to conduct similar analyses on samples from patients infected with currently circulating variants. This is particularly important given that the entire population is either vaccinated or has previously had the disease. Therefore, metabolic responses in the current patient cohort may differ from those in individuals recruited during the early pandemic. Nevertheless, metabolic profiling in plasma and BALF depicts a comprehensive view of host metabolic alterations in COVID-19 patients with varying severity. The identification of the critical metabolite indoxyl sulfate offers a potentially applicable biomarker for clinical use and advances our understanding of COVID-19 immunopathogenesis.

## Figures and Tables

**Table 1:** Study participants’ demographics.

Variables	Control (*n* = 3)	Moderate COVID-19 (n = 13)	Severe COVID-19 (*n* = 16)
**Sex—n (%)**			
Male	2 (66.7)	6 (46.2)	5 (31.2)
Female	1 (33.3)	7 (53.8)	11 (68.8)
**Age—year**			
Mean ± SD	36.7 (8.9)	67.1 (17.8)	62.4 (12.3)
Median (IQR)	42 (10)	66 (30)	64 (10.5)
Range	24–44	40–95	43–80
**BMI**			
Mean ± SD	38.48 (6.05)	28.45 (7.15)	35.41 (9.52)
Median (IQR)	35.15 (6.83)	27.11 (13)	38.35 (13.67)
Range	33.31–46.97	20.4–40.59	21.68–53.81
**Ethnicity—*n* (%)**			
AA	1 (33)	8 (42.6)	4 (25)
White	1 (33)	5 (38.4)	11 (68.8)
Hispanic/Others	1 (33)	0 (0)	1 (6.2)
**Comorbidity—*n* (%)**			
Hypertension	0 (0)	10 (76.9)	10 (62.5)
Diabetes	0 (0)	4 (30.8)	10 (62.5)
Respiratory Disease	0 (0)	2 (15.4)	10 (62.5)
Cardiac Disease	0 (0)	3 (23.1)	7 (43.8)
Kidney Disease	0 (0)	1 (7.7)	6 (37.5)

## Data Availability

The original contributions presented in this study are included in the article/Supplementary Material. Further inquiries can be directed to the corresponding authors.

## Supplementary Materials

The following supporting information can be downloaded at: https://www.mdpi.com/article/10.3390/cells14040256/s1. Figure S1. BALF metabolome remodeling due to severity of SARS-CoV-2 infection. Figure S2. t-SNE plot analysis. Table S1. Metabolites quantified in plasma samples. Table S2. Metabolites quantified in BALF samples. Table S3. Correlative analysis between adundance of different mitabolites and D-dimer, Ferritin, C-reactive protein, and different cytokines.
