# Supplementary material for: Metabolomics Profiling Reveals Critical Roles of Indoxyl Sulfate in the Regulation of Innate Monocytes in COVID-19
Source: Cells. 2025 Feb 11;14(4):256. doi: 10.3390/cells14040256 (PMC11853107; doi:10.3390/cells14040256)
Supplement: Supplementary file 1 [file cells-14-00256-s001.zip › cells-3447148-supplementary.pdf]

**Supplemental Figure 1**

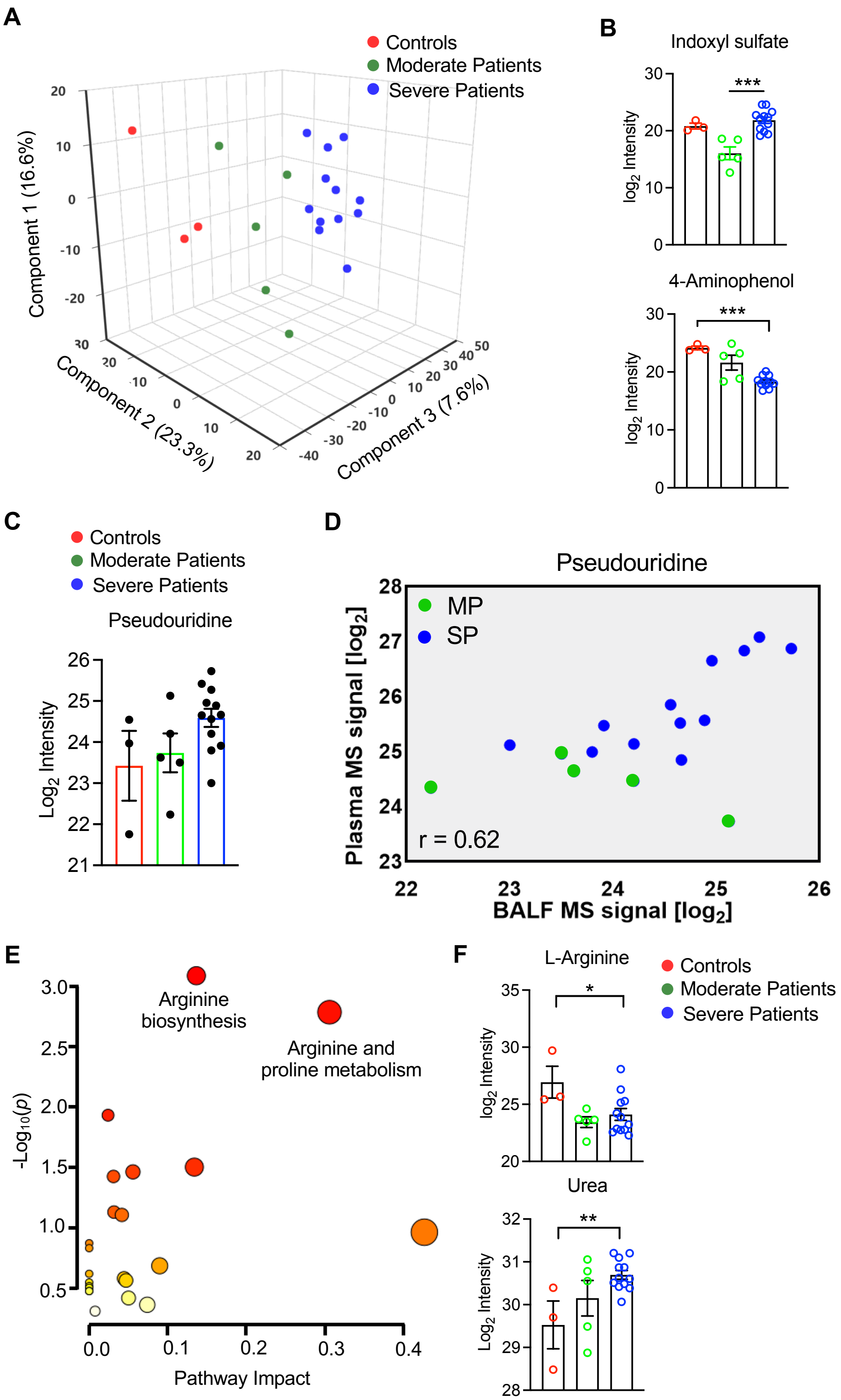


**Fig. S1. BALF metabolome remodeling due to severity of SARS-CoV-2 infection.** (A) 3D score plot of PLS-DA analysis using all metabolites detected in BALF by 2DLC-MS under positive and negative modes. (B) Quantification results of two significantly changed metabolites in BALF. One-way ANOVA was used for statistical significance test with Tukey method for the post-hoc analysis with multiple test correction: *** $q < 0.001$. (C) The abundance change of pseudouridine and 2-furoic acid in BALF detected by untargeted metabolomics using 2DLC-MS. (D) The Spearman's rank correlation of pseudouridine and 2-furoic acid in paired BALF and plasma samples. One-way ANOVA was used for statistical significance test with Tukey method for post-hoc analysis and Benjamini and Hochberg method for multiple test correction. (E) Pathway analysis using all significantly changed metabolites in BALF. (F) Quantification results of arginine and urea in BALF. One-way ANOVA was used for statistical significance test with Tukey method for the post-hoc analysis without multiple test correction: * $p < 0.05$; ** $p < 0.01$.

**Supplemental Figure 2**

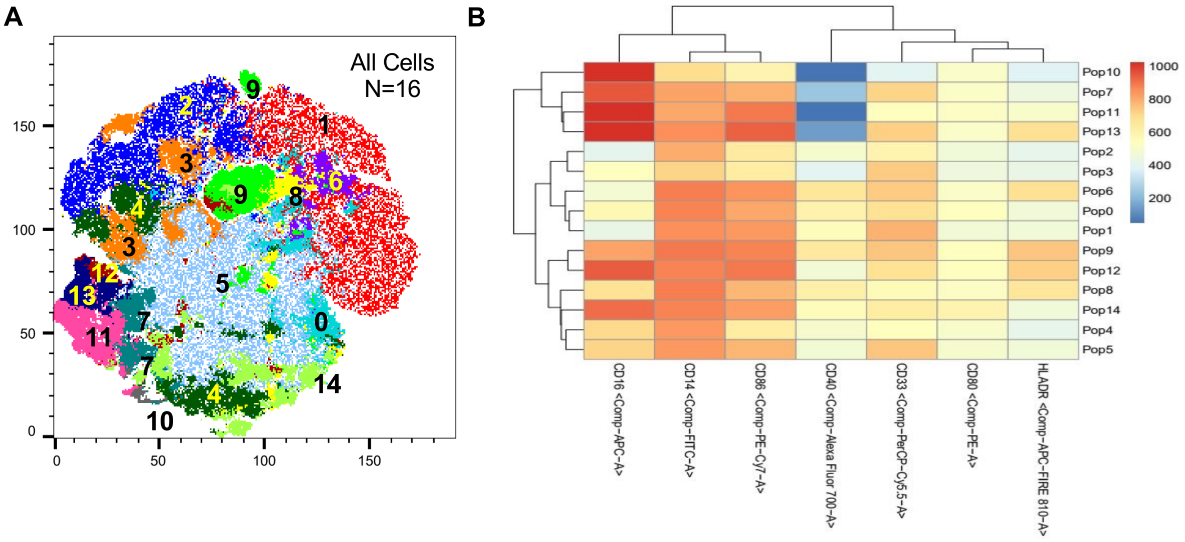


**Fig. S2. t-SNE plot analysis.** (A) t-SNE plot for all cells. (B) Heatmap for markers expression on different myeloid cell populations.

Table S1. Metabolites quantified in plasma samples

| | | Negative | | | | | |
|---|---|---|---|---|---|---|---|
| | | MP vs HD | | SP vs MP | | SP vs HD | |
| | Compound Name | q_value | FC | q_value | FC | q_value | FC |
| Significantly changed metabolites | 16-Hydroxy hexadecanoic acid | 0.999 | 1.120 | < 0.001 | 0.530 | 0.017 | 0.594 |
| | 2-Furoic acid | < 0.001 | 0.038 | 0.901 | 0.971 | < 0.001 | 0.037 |
| | 2-Hydroxy-2-methylbutyric acid | < 0.001 | 9.564 | 0.073 | 0.853 | < 0.001 | 8.160 |
| | 2-Hydroxyvaleric acid | < 0.001 | 3.156 | 0.115 | 0.665 | 0.012 | 2.097 |
| | 2-Phosphoglyceric acid | 0.009 | 3.722 | 0.683 | 1.395 | < 0.001 | 5.190 |
| | 3,3-Dimethylglutaric acid | 0.253 | 0.671 | 0.021 | 0.630 | < 0.001 | 0.423 |
| | 3-Methyladipic acid | 0.253 | 1.252 | 0.044 | 1.424 | < 0.001 | 1.783 |
| | Acrylic acid | < 0.001 | 0.306 | 0.145 | 1.129 | < 0.001 | 0.345 |
| | Alpha-Lactose | < 0.001 | 0.102 | < 0.001 | 1.183 | < 0.001 | 0.353 |
| | Alpha-N-Phenylacetyl-L-glutamine | 0.294 | 5.289 | 0.092 | 3.467 | 0.006 | 14.687 |
| | Ascorbic acid | < 0.001 | 0.018 | 1.000 | 2.777 | < 0.001 | 0.026 |
| | Beta-D-Glucopyranuronic acid | 0.073 | 2.988 | < 0.001 | 2.819 | < 0.001 | 8.422 |
| | cis-Aconitic acid | < 0.001 | 0.183 | 0.997 | 0.969 | < 0.001 | 0.177 |
| | Citric acid | < 0.001 | 0.015 | 0.584 | 0.907 | < 0.001 | 0.014 |
| | D-Glucose | < 0.001 | 0.301 | 0.288 | 1.180 | < 0.001 | 0.355 |
| | D-Mannose | < 0.001 | 0.280 | 0.602 | 1.326 | < 0.001 | 0.372 |
| | Hydroxyphenyllactic acid | 0.143 | 1.974 | 0.288 | 1.574 | 0.006 | 3.106 |
| | Indoxyl sulfate | 0.576 | 0.838 | 0.049 | 2.916 | 0.928 | 2.444 |
| | Inosine | < 0.001 | 1.460 | 0.283 | 0.041 | < 0.001 | 0.059 |
| | Isocitric acid | < 0.001 | 0.032 | 0.288 | 0.887 | < 0.001 | 0.029 |
| | Itaconic acid | < 0.001 | 0.229 | 1.000 | 0.941 | < 0.001 | 0.215 |
| | Glycolic acid | < 0.001 | 0.606 | < 0.001 | 0.762 | < 0.001 | 0.462 |
| | L-Aspartic acid | < 0.001 | 8.353 | 1.000 | 0.984 | < 0.001 | 8.221 |
| | Levulinic acid | 0.169 | 1.992 | 0.941 | 1.045 | 0.049 | 2.081 |
| | L-Glyceric acid | 0.018 | 0.593 | 0.226 | 0.668 | < 0.001 | 0.396 |
| | L-Lactic acid | < 0.001 | 4.459 | 1.000 | 1.002 | < 0.001 | 4.467 |
| | L-Malic acid | 0.298 | 0.354 | 0.785 | 1.003 | 0.049 | 0.355 |
| | Malonic acid | < 0.001 | 0.483 | 1.000 | 1.033 | < 0.001 | 0.499 |
| | Methanesulfonic acid | 0.177 | 1.756 | 0.385 | 1.219 | 0.012 | 2.140 |
| | Methylsuccinic acid | < 0.001 | 0.346 | 0.837 | 1.149 | < 0.001 | 0.397 |
| | N-Acetylglutamate | 0.004 | 3.358 | 1.000 | 1.128 | < 0.001 | 3.788 |
| | N-Acetyl-L-alanine | 0.007 | 2.547 | 0.977 | 1.199 | 0.003 | 3.054 |
| | Orotidine | 0.873 | 15.367 | 0.062 | 4.141 | 0.021 | 63.628 |
| | Oxalic acid | 0.007 | 13.257 | 1.000 | 0.405 | 0.014 | 5.373 |
| | Pseudouridine | 0.004 | 3.415 | 0.021 | 1.640 | < 0.001 | 5.601 |
| | Pyroglutamic acid | < 0.001 | 8.151 | 1.000 | 0.293 | < 0.001 | 2.390 |
| | Ribonolactone | < 0.001 | 0.069 | 1.000 | 1.013 | < 0.001 | 0.070 |
| | Suberic acid | 0.027 | 1.958 | 1.000 | 0.966 | 0.008 | 1.892 |
| | Succinic acid semialdehyde | < 0.001 | 2.140 | 0.014 | 0.623 | 0.363 | 1.334 |
| | Taurine | < 0.001 | 0.355 | 1.000 | 1.166 | < 0.001 | 0.413 |
| | Uric acid | 0.073 | 1.426 | 1.000 | 0.937 | 0.047 | 1.336 |
| | Xanthine | < 0.001 | 4.914 | 0.508 | 1.789 | 0.010 | 8.793 |
| | Xanthosine | 0.009 | 73.187 | 0.246 | 1.087 | < 0.001 | 79.559 |
| Unchanged metabolites | 12-Hydroxydodecanoic acid | > 0.05 | 0.985 | > 0.05 | 0.821 | > 0.05 | 0.809 |
| | 1H-Indole-3-carboxaldehyde | > 0.05 | 0.668 | > 0.05 | 0.895 | > 0.05 | 0.598 |
| | 3-Hydroxybutyric acid | > 0.05 | 6.555 | > 0.05 | 0.565 | > 0.05 | 3.703 |
| | 4-Dodecylbenzenesulfonic acid Na salt | > 0.05 | 1.319 | > 0.05 | 1.079 | > 0.05 | 1.424 |
| | 4-Hydroxyproline | > 0.05 | 1.541 | > 0.05 | 0.706 | > 0.05 | 1.089 |
| | 2-Hydroxybutyric acid | > 0.05 | 5.401 | > 0.05 | 0.917 | > 0.05 | 4.951 |
| | Acetaminophen glucuronide | > 0.05 | 157.226 | > 0.05 | 0.266 | > 0.05 | 41.832 |
| | D-2-Hydroxyglutaric acid | > 0.05 | 0.642 | > 0.05 | 1.131 | > 0.05 | 0.727 |
| | Fumarate | > 0.05 | 0.934 | > 0.05 | 0.935 | 0.029 | 0.873 |
| | Succinic acid | 0.004 | 0.807 | > 0.05 | 1.165 | 0.049 | 0.941 |
| | Glucaric acid | > 0.05 | 2.826 | > 0.05 | 1.688 | > 0.05 | 4.769 |
| | Hippuric acid | > 0.05 | 0.611 | > 0.05 | 6.569 | > 0.05 | 4.014 |
| | Indolelactic acid | > 0.05 | 0.665 | > 0.05 | 1.537 | > 0.05 | 1.023 |
| | Isobutyric acid | > 0.05 | 1.645 | > 0.05 | 1.939 | > 0.05 | 3.190 |
| | L-Glutamine | > 0.05 | 0.917 | > 0.05 | 0.937 | > 0.05 | 0.860 |
| | L-Histidine | > 0.05 | 0.779 | > 0.05 | 1.018 | > 0.05 | 0.793 |
| | L-Methionine | > 0.05 | 0.785 | > 0.05 | 1.066 | > 0.05 | 0.837 |
| | L-Phenylalanine | > 0.05 | 1.192 | > 0.05 | 1.097 | > 0.05 | 1.308 |
| | Kynurenic acid | > 0.05 | 1.753 | > 0.05 | 1.439 | > 0.05 | 2.521 |
| | p-Cresol | > 0.05 | 0.986 | > 0.05 | 2.443 | > 0.05 | 2.408 |
| | L-Alanine | > 0.05 | 0.658 | > 0.05 | 3.824 | > 0.05 | 2.517 |
| | L-Arginine | > 0.05 | 0.517 | > 0.05 | 1.097 | > 0.05 | 0.568 |
| | L-Cystine | > 0.05 | 2.699 | > 0.05 | 0.900 | > 0.05 | 2.428 |
| | L-Threonine | > 0.05 | 0.486 | > 0.05 | 1.354 | > 0.05 | 0.658 |
| | L-Tryptophan | > 0.05 | 0.453 | > 0.05 | 0.998 | > 0.05 | 0.452 |
| | N-Acetylisoleucine | > 0.05 | 1.084 | > 0.05 | 1.747 | > 0.05 | 1.893 |
| | Oxoglutaric acid | > 0.05 | 0.801 | > 0.05 | 0.977 | > 0.05 | 0.782 |
| | Pantothenic acid | > 0.05 | 1.079 | > 0.05 | 1.206 | > 0.05 | 1.302 |
| | Phenyllactic acid | > 0.05 | 1.767 | > 0.05 | 1.337 | > 0.05 | 2.362 |
| | Pyruvic acid | > 0.05 | 1.543 | > 0.05 | 1.031 | > 0.05 | 1.591 |
| | 1-Methyl-L-histidine/3-Methyl-L-histidine | > 0.05 | 0.340 | > 0.05 | 0.601 | > 0.05 | 0.204 |
| | 2-Hydroxymyristic acid | > 0.05 | 0.812 | > 0.05 | 0.969 | > 0.05 | 0.787 |
| | 3-Hydroxycapric acid | > 0.05 | 1.045 | > 0.05 | 0.882 | > 0.05 | 0.921 |
| | 3-Hydroxyisovaleric acid | > 0.05 | 2.737 | > 0.05 | 1.106 | > 0.05 | 3.028 |
| | 3-Hydroxymethylglutaric acid | > 0.05 | 1.209 | > 0.05 | 1.122 | > 0.05 | 1.357 |
| | 3-Methyladipic acid | > 0.05 | 1.235 | > 0.05 | 1.341 | > 0.05 | 1.656 |
| | 4-Hydroxybenzaldehyde | > 0.05 | 0.946 | > 0.05 | 1.127 | > 0.05 | 1.066 |
| | 4-Oxoproline | > 0.05 | 1.670 | > 0.05 | 0.433 | > 0.05 | 0.723 |
| | 6-Hydroxyhexanoic acid | > 0.05 | 1.322 | > 0.05 | 0.732 | > 0.05 | 0.968 |
| | Adipic acid | > 0.05 | 1.130 | > 0.05 | 1.759 | > 0.05 | 1.989 |
| | Citraconic acid | > 0.05 | 1.089 | > 0.05 | 0.960 | > 0.05 | 1.046 |
| | Citrulline | > 0.05 | 0.881 | > 0.05 | 1.009 | > 0.05 | 0.889 |
| | L-Glutamic acid | > 0.05 | 1.290 | > 0.05 | 1.687 | > 0.05 | 2.176 |
| | L-Serine | > 0.05 | 0.951 | > 0.05 | 0.801 | > 0.05 | 0.762 |
| | L-Tyrosine | > 0.05 | 0.728 | > 0.05 | 1.218 | > 0.05 | 0.887 |
| | Phosphoenolpyruvic acid | > 0.05 | 3.325 | > 0.05 | 2.228 | > 0.05 | 7.410 |
| | Saccharin | > 0.05 | 0.720 | > 0.05 | 1.750 | > 0.05 | 1.259 |
| | Tartaric acid | > 0.05 | 0.899 | > 0.05 | 1.507 | > 0.05 | 1.355 |
| | Azelaic acid | > 0.05 | 1.050 | > 0.05 | 0.932 | > 0.05 | 0.979 |
| | Ornithine | > 0.05 | 0.713 | > 0.05 | 1.540 | > 0.05 | 1.098 |

| | | Positive | | | | | |
|---|---|---|---|---|---|---|---|
| | | MP vs HD | | SP vs MP | | SP vs HD | |
| | Compound Name | q_value | FC | q_value | FC | q_value | FC |
| Significantly changed metabolites | 1-Methy-L-lhistidine/3-Methyl-L-histidine | < 0.001 | 3.035 | 1.000 | 1.228 | < 0.001 | 3.726 |
| | 2-Hydroxycinnamic acid | < 0.001 | 0.446 | 0.021 | 1.462 | 0.151 | 0.651 |
| | 3,4-Dihydroxyhydrocinnamic acid | 0.004 | 1.492 | 1.000 | 0.997 | 0.003 | 1.487 |
| | 4-Hydroxybenzaldehyde | 0.004 | 0.516 | 0.941 | 1.273 | 0.010 | 0.657 |
| | 6-Pentyl-2H-pyran-2-one | 0.999 | 1.090 | 0.014 | 1.530 | 0.282 | 1.668 |
| | Adenosine | < 0.001 | 0.002 | 0.068 | 2.185 | < 0.001 | 0.004 |
| | Choline | 0.999 | 0.967 | < 0.001 | 2.233 | 0.088 | 2.159 |
| | Citrulline | 0.012 | 0.492 | 1.000 | 0.871 | 0.008 | 0.429 |
| | Indole-3-propionic acid | < 0.001 | 0.442 | 1.000 | 1.039 | < 0.001 | 0.459 |
| | L-Acetylcarnitine | 0.018 | 2.179 | 1.000 | 0.992 | 0.044 | 2.162 |
| | L-Alanine | 0.025 | 0.621 | 1.000 | 0.909 | 0.006 | 0.564 |
| | L-Glutamine | < 0.001 | 0.234 | 1.000 | 0.968 | < 0.001 | 0.226 |
| | L-Tyrosine | 0.004 | 0.583 | 0.977 | 1.160 | 0.012 | 0.676 |
| | Methacholine | 0.020 | 2.136 | 0.678 | 1.590 | < 0.001 | 3.395 |
| | Methylimidazoleacetic acid | 0.337 | 3.028 | 0.038 | 3.037 | 0.003 | 9.196 |
| | N6,N6,N6-Trimethyl-L-lysine | 0.400 | 1.512 | < 0.001 | 1.829 | < 0.001 | 2.765 |
| | Ornithine | 0.297 | 1.488 | < 0.001 | 1.299 | < 0.001 | 1.933 |
| | Symmetric dimethylarginine | 0.174 | 1.744 | 0.684 | 1.226 | 0.023 | 2.138 |
| | Triethanolamine | 0.999 | 0.994 | 0.014 | 0.769 | 0.068 | 0.764 |
| | Trigonelline | < 0.001 | 0.130 | 0.723 | 0.731 | < 0.001 | 0.095 |
| | Urea | 0.075 | 1.751 | 0.114 | 1.347 | < 0.001 | 2.359 |
| Unchanged metabolites | 3-Methylhistidine | > 0.05 | 1.721 | > 0.05 | 0.465 | > 0.05 | 0.800 |
| | 3-(4-Methoxyphenyl)-2-propenal | > 0.05 | 1.113 | > 0.05 | 1.076 | > 0.05 | 1.197 |
| | 4-Ethylbenzaldehyde | > 0.05 | 0.900 | > 0.05 | 0.906 | > 0.05 | 0.815 |
| | 6-Methylnicotinamide | > 0.05 | 0.579 | > 0.05 | 1.835 | > 0.05 | 1.063 |
| | Dibutyl phthalate | > 0.05 | 1.005 | > 0.05 | 0.994 | > 0.05 | 0.999 |
| | L-Glutamic acid | > 0.05 | 1.014 | > 0.05 | 1.126 | > 0.05 | 1.141 |
| | L-Hypoglycin A | > 0.05 | 0.953 | > 0.05 | 1.049 | > 0.05 | 1.000 |
| | L-Proline | > 0.05 | 0.785 | > 0.05 | 1.169 | > 0.05 | 0.918 |
| | L-Threonine | > 0.05 | 0.823 | > 0.05 | 1.048 | > 0.05 | 0.862 |
| | 5-Hydroxyindoleacetic acid | > 0.05 | 1.853 | > 0.05 | 1.684 | > 0.05 | 3.120 |
| | Acetaminophen | > 0.05 | 30.311 | > 0.05 | 0.133 | > 0.05 | 4.037 |
| | Alpha-N-Phenylacetyl-L-glutamine | > 0.05 | 1.493 | > 0.05 | 2.168 | > 0.05 | 3.238 |
| | Creatine | > 0.05 | 0.743 | > 0.05 | 2.343 | > 0.05 | 1.740 |
| | L-Aspartic acid | > 0.05 | 1.110 | > 0.05 | 1.291 | > 0.05 | 1.433 |
| | L-Methionine | > 0.05 | 0.279 | > 0.05 | 1.147 | > 0.05 | 0.320 |
| | L-Serine | > 0.05 | 0.815 | > 0.05 | 0.760 | > 0.05 | 0.619 |
| | L-Tryptophan | > 0.05 | 0.381 | > 0.05 | 1.273 | > 0.05 | 0.485 |
| | D-Glucose | > 0.05 | 1.282 | > 0.05 | 1.470 | > 0.05 | 1.885 |
| | D-Maltose | > 0.05 | 0.354 | > 0.05 | 10.537 | > 0.05 | 3.728 |
| | Glutarylcarnitine | > 0.05 | 0.943 | > 0.05 | 2.023 | > 0.05 | 1.907 |
| | Hippuric acid | > 0.05 | 0.265 | > 0.05 | 7.478 | > 0.05 | 1.979 |
| | Hypoxanthine | > 0.05 | 1.444 | > 0.05 | 0.957 | > 0.05 | 1.381 |
| | N8-Acetylspermidine | > 0.05 | 2.065 | > 0.05 | 1.385 | > 0.05 | 2.860 |
| | Pipecolic acid | > 0.05 | 0.542 | > 0.05 | 0.501 | > 0.05 | 0.272 |
| | Proline betaine | > 0.05 | 5.108 | > 0.05 | 0.062 | > 0.05 | 0.319 |
| | Taurine | > 0.05 | 0.423 | > 0.05 | 0.902 | > 0.05 | 0.381 |
| | N-Acetylarylamine | > 0.05 | 0.583 | > 0.05 | 0.712 | > 0.05 | 0.416 |
| | Oleamide | > 0.05 | 0.786 | > 0.05 | 1.287 | > 0.05 | 1.012 |
| | Sulfamethoxazole | > 0.05 | 1.152 | > 0.05 | 1.975 | > 0.05 | 2.275 |
| | o-Tyrosine | > 0.05 | 1.043 | > 0.05 | 2.117 | > 0.05 | 2.208 |
| | 1-Methyladenosine | > 0.05 | 1.376 | > 0.05 | 0.988 | > 0.05 | 1.359 |
| | 2-Piperidinone | > 0.05 | 0.491 | > 0.05 | 0.696 | > 0.05 | 0.342 |
| | Acetylcholine | > 0.05 | 0.625 | > 0.05 | 1.429 | > 0.05 | 0.893 |
| | Betaine | > 0.05 | 1.076 | > 0.05 | 1.089 | > 0.05 | 1.172 |
| | Creatinine | > 0.05 | 1.018 | > 0.05 | 1.496 | > 0.05 | 1.522 |
| | Ecgonine | > 0.05 | 1.539 | > 0.05 | 1.056 | > 0.05 | 1.626 |
| | L-Alpha-aminobutyric acid | > 0.05 | 1.310 | > 0.05 | 0.968 | > 0.05 | 1.267 |
| | L-Arginine | > 0.05 | 0.661 | > 0.05 | 1.042 | > 0.05 | 0.688 |
| | L-Carnitine | > 0.05 | 1.392 | > 0.05 | 1.062 | > 0.05 | 1.478 |
| | L-Cystine | > 0.05 | 1.634 | > 0.05 | 0.752 | > 0.05 | 1.229 |
| | L-Histidine | > 0.05 | 1.401 | > 0.05 | 0.818 | > 0.05 | 1.146 |
| | L-Isoleucine | > 0.05 | 1.116 | > 0.05 | 0.912 | > 0.05 | 1.019 |
| | L-Leucine | > 0.05 | 0.991 | > 0.05 | 0.934 | > 0.05 | 0.925 |
| | L-Phenylalanine | > 0.05 | 1.198 | > 0.05 | 1.099 | > 0.05 | 1.317 |
| | N,N-Dimethylaniline | > 0.05 | 1.017 | > 0.05 | 1.052 | > 0.05 | 1.070 |
| | Octadecanamide | > 0.05 | 0.963 | > 0.05 | 0.986 | > 0.05 | 0.950 |
| | Palmitic amide | > 0.05 | 1.028 | > 0.05 | 0.964 | > 0.05 | 0.991 |
| | Penbutolol | > 0.05 | 0.598 | > 0.05 | 1.024 | > 0.05 | 0.613 |
| | Propionylcarnitine | > 0.05 | 1.067 | > 0.05 | 2.394 | > 0.05 | 2.553 |
| | Tranexamic acid | > 0.05 | 0.935 | > 0.05 | 0.823 | > 0.05 | 0.769 |
| | L-Proline | > 0.05 | 0.697 | > 0.05 | 1.215 | > 0.05 | 0.847 |
| | N-Acetylornithine | > 0.05 | 0.492 | > 0.05 | 0.962 | > 0.05 | 0.473 |

Table S2. Metabolites quantified in BALF samples

| | Compound Name | MP vs. HD q-Value | MP vs. HD FC | SP vs. HD q-Value | SP vs. HD FC | SP vs. MP q-Value | SP vs. MP FC | Platforms |
|---|---|---|---|---|---|---|---|---|
| Significantly changed metabolites | Indoxyl sulfate | > 0.05 | 0.08 | > 0.05 | 3.55 | 0.000 | 44.61 | Negative |
| | 4-Aminophenol | > 0.05 | 0.50 | 0.000 | 0.02 | > 0.05 | 0.04 | Positive |
| Unchanged metabolites | 1H-Indole-3-carboxaldehyde | > 0.05 | 1.19 | > 0.05 | 0.90 | > 0.05 | 0.75 | Negative |
| | 2-Furoic acid | > 0.05 | 1.98 | > 0.05 | 6.68 | > 0.05 | 3.37 | Negative |
| | 2-Hydroxybutyric acid | > 0.05 | 1.34 | > 0.05 | 2.42 | > 0.05 | 1.81 | Negative |
| | 2-Hydroxyvaleric acid | > 0.05 | 0.61 | > 0.05 | 1.24 | > 0.05 | 2.03 | Negative |
| | 2-Methyl-3-hydroxybutyric acid | > 0.05 | 1.58 | > 0.05 | 1.49 | > 0.05 | 0.94 | Negative |
| | 3,3-Dimethylglutaric acid | > 0.05 | 1.70 | > 0.05 | 0.87 | > 0.05 | 0.51 | Negative |
| | 3-Hydroxybutyric acid | > 0.05 | 1.13 | > 0.05 | 0.90 | > 0.05 | 0.80 | Negative |
| | 3-Methylxanthine | > 0.05 | 0.14 | > 0.05 | 0.44 | > 0.05 | 3.02 | Negative |
| | 4-Amino-5-aminomethyl-2-methylpyrimidine | > 0.05 | 0.79 | > 0.05 | 1.23 | > 0.05 | 1.56 | Negative |
| | 4-Dodecylbenzenesulfonic acid Na salt | > 0.05 | 1.66 | > 0.05 | 1.07 | > 0.05 | 0.65 | Negative |
| | 4-Hydroxybenzaldehyde | > 0.05 | 0.94 | > 0.05 | 0.62 | > 0.05 | 0.66 | Negative |
| | 4-Hydroxyproline | > 0.05 | 0.03 | > 0.05 | 0.02 | > 0.05 | 0.52 | Negative |
| | 4-Oxoproline | > 0.05 | 0.01 | > 0.05 | 0.02 | > 0.05 | 1.93 | Negative |
| | 5-Hydroxy-L-tryptophan | > 0.05 | 0.12 | > 0.05 | 0.48 | > 0.05 | 3.89 | Negative |
| | 5-Sulfosalicylic acid | > 0.05 | 1.51 | > 0.05 | 6.22 | > 0.05 | 4.13 | Negative |
| | 6-Hydroxyhexanoic acid | > 0.05 | 0.42 | > 0.05 | 0.27 | > 0.05 | 0.65 | Negative |
| | Acrylic acid | > 0.05 | 0.12 | > 0.05 | 0.13 | > 0.05 | 1.09 | Negative |
| | Adenine | > 0.05 | 0.20 | > 0.05 | 0.16 | > 0.05 | 0.80 | Negative |
| | Adipic acid | > 0.05 | 1.95 | > 0.05 | 1.63 | > 0.05 | 0.83 | Negative |
| | Alpha-N-Phenylacetyl-L-glutamine | > 0.05 | 0.41 | > 0.05 | 20.89 | > 0.05 | 50.72 | Negative |
| | Azelaic acid | > 0.05 | 1.35 | > 0.05 | 0.96 | > 0.05 | 0.71 | Negative |
| | Benzoic acid | > 0.05 | 0.96 | > 0.05 | 0.48 | > 0.05 | 0.51 | Negative |
| | Beta-Leucine | > 0.05 | 0.18 | > 0.05 | 0.54 | > 0.05 | 2.98 | Negative |
| | But-2-enoic acid | > 0.05 | 1.04 | > 0.05 | 1.06 | > 0.05 | 1.01 | Negative |
| | cis-Aconitic acid | > 0.05 | 1.24 | > 0.05 | 1.14 | > 0.05 | 0.92 | Negative |
| | Citric acid | > 0.05 | 1.12 | > 0.05 | 3.28 | > 0.05 | 2.91 | Negative |
| | D-Fructose | > 0.05 | 0.37 | > 0.05 | 0.27 | > 0.05 | 0.74 | Negative |
| | D-Glucose | > 0.05 | 0.31 | > 0.05 | 0.33 | > 0.05 | 1.06 | Negative |
| | D-Glyceraldehyde 3-phosphate | > 0.05 | 1.74 | > 0.05 | 0.77 | > 0.05 | 0.44 | Negative |
| | Dibutyl phthalate | > 0.05 | 1.01 | > 0.05 | 1.04 | > 0.05 | 1.03 | Negative |
| | Dihydroxyacetone phosphate | > 0.05 | 1.34 | > 0.05 | 0.78 | > 0.05 | 0.58 | Negative |
| | D-Phenyllactic acid | > 0.05 | 1.55 | > 0.05 | 0.51 | > 0.05 | 0.33 | Negative |
| | Fumaric acid | > 0.05 | 1.02 | > 0.05 | 1.09 | > 0.05 | 1.06 | Negative |
| | Gluconic acid | > 0.05 | 0.04 | > 0.05 | 0.04 | > 0.05 | 0.85 | Negative |
| | Glucose 6-phosphate | > 0.05 | 0.08 | > 0.05 | 0.25 | > 0.05 | 3.12 | Negative |
| | Guanosine | > 0.05 | 1.08 | > 0.05 | 0.54 | > 0.05 | 0.50 | Negative |
| | Hexanoylglycine | > 0.05 | 0.57 | > 0.05 | 0.47 | > 0.05 | 0.83 | Negative |
| | Hippuric acid | > 0.05 | 0.98 | > 0.05 | 6.19 | > 0.05 | 6.33 | Negative |
| | Hydroxyphenyllactic acid | > 0.05 | 2.73 | > 0.05 | 4.80 | > 0.05 | 1.76 | Negative |
| | Hypoxanthine | > 0.05 | 0.43 | > 0.05 | 0.65 | > 0.05 | 1.51 | Negative |
| | Indolelactic acid | > 0.05 | 2.20 | > 0.05 | 4.25 | > 0.05 | 1.93 | Negative |
| | Inosine | > 0.05 | 0.47 | > 0.05 | 0.79 | > 0.05 | 1.69 | Negative |
| | Isocitric acid | > 0.05 | 1.76 | > 0.05 | 3.31 | > 0.05 | 1.88 | Negative |
| | Itaconic acid | > 0.05 | 1.02 | > 0.05 | 1.49 | > 0.05 | 1.47 | Negative |
| | Ketoleucine | > 0.05 | 1.22 | > 0.05 | 1.33 | > 0.05 | 1.09 | Negative |
| | L-Arginine | > 0.05 | 0.04 | > 0.05 | 0.05 | > 0.05 | 1.15 | Negative |
| | L-Cystine | > 0.05 | 0.07 | > 0.05 | 0.14 | > 0.05 | 2.08 | Negative |
| | L-Dopa | > 0.05 | 0.14 | > 0.05 | 0.72 | > 0.05 | 5.10 | Negative |
| | Levulinic acid | > 0.05 | 1.69 | > 0.05 | 1.68 | > 0.05 | 1.00 | Negative |
| | L-Glutamine | > 0.05 | 0.17 | > 0.05 | 0.39 | > 0.05 | 2.28 | Negative |
| | L-Histidine | > 0.05 | 0.29 | > 0.05 | 0.88 | > 0.05 | 3.03 | Negative |
| | L-Isoleucine | > 0.05 | 0.09 | > 0.05 | 0.55 | > 0.05 | 6.38 | Negative |
| | L-Lactic acid | > 0.05 | 1.61 | > 0.05 | 2.02 | > 0.05 | 1.26 | Negative |
| | L-Leucine | > 0.05 | 0.26 | > 0.05 | 0.86 | > 0.05 | 3.31 | Negative |
| | L-Lysine | > 0.05 | 0.35 | > 0.05 | 2.25 | > 0.05 | 6.34 | Negative |
| | L-Malic acid | > 0.05 | 0.25 | > 0.05 | 0.44 | > 0.05 | 1.79 | Negative |
| | L-Methionine | > 0.05 | 0.10 | > 0.05 | 0.41 | > 0.05 | 3.96 | Negative |
| | L-Phenylalanine | > 0.05 | 0.44 | > 0.05 | 1.25 | > 0.05 | 2.85 | Negative |
| | L-Threonine | > 0.05 | 0.49 | > 0.05 | 1.26 | > 0.05 | 2.56 | Negative |
| | Methylglutaric acid | > 0.05 | 1.52 | > 0.05 | 0.65 | > 0.05 | 0.43 | Negative |
| | Methylglutaric acid | > 0.05 | 1.52 | > 0.05 | 0.78 | > 0.05 | 2.56 | Negative |
| | Monoethylhexyl phthalic acid | > 0.05 | 0.81 | > 0.05 | 0.21 | > 0.05 | 0.26 | Negative |
| | N-Acetylglutamic acid | > 0.05 | 0.79 | > 0.05 | 0.75 | > 0.05 | 0.94 | Negative |
| | N-Acetylglutamine | > 0.05 | 0.88 | > 0.05 | 1.07 | > 0.05 | 1.22 | Negative |
| | N-Acetyl-L-aspartic acid | > 0.05 | 0.40 | > 0.05 | 0.41 | > 0.05 | 1.01 | Negative |
| | N-Acetyl-L-methionine | > 0.05 | 1.07 | > 0.05 | 7.92 | > 0.05 | 7.39 | Negative |
| | N-Alpha-acetyllysine | > 0.05 | 4.26 | > 0.05 | 14.46 | > 0.05 | 3.40 | Negative |
| | Ornithine | > 0.05 | 0.09 | > 0.05 | 0.14 | > 0.05 | 1.52 | Negative |
| | Oxoglutaric acid | > 0.05 | 2.24 | > 0.05 | 2.32 | > 0.05 | 1.04 | Negative |
| | Pantothenic acid | > 0.05 | 0.08 | > 0.05 | 0.27 | > 0.05 | 3.39 | Negative |
| | Picolinic acid | > 0.05 | 0.33 | > 0.05 | 0.29 | > 0.05 | 0.87 | Negative |
| | Pseudouridine | > 0.05 | 1.16 | > 0.05 | 1.92 | > 0.05 | 1.66 | Negative |
| | Pyroglutamic acid | > 0.05 | 0.10 | > 0.05 | 0.10 | > 0.05 | 0.98 | Negative |
| | Pyruvic acid | > 0.05 | 1.09 | > 0.05 | 0.86 | > 0.05 | 0.79 | Negative |
| | Saccharin | > 0.05 | 2.52 | > 0.05 | 3.56 | > 0.05 | 1.41 | Negative |
| | Salicylic acid | > 0.05 | 1.27 | > 0.05 | 1.58 | > 0.05 | 1.24 | Negative |
| | Suberic acid | > 0.05 | 1.29 | > 0.05 | 0.92 | > 0.05 | 0.71 | Negative |
| | Succinic acid | > 0.05 | 1.23 | > 0.05 | 1.44 | > 0.05 | 1.17 | Negative |
| | Taurine | > 0.05 | 0.73 | > 0.05 | 1.22 | > 0.05 | 1.67 | Negative |
| | Theophylline | > 0.05 | 0.07 | > 0.05 | 0.03 | > 0.05 | 0.38 | Negative |
| | Uracil | > 0.05 | 0.36 | > 0.05 | 0.49 | > 0.05 | 1.37 | Negative |
| | Uric acid | > 0.05 | 0.73 | > 0.05 | 0.77 | > 0.05 | 1.06 | Negative |
| | Uridine | > 0.05 | 0.39 | > 0.05 | 0.59 | > 0.05 | 1.53 | Negative |
| | Xanthine | > 0.05 | 0.74 | > 0.05 | 1.02 | > 0.05 | 1.37 | Negative |
| | Xanthosine | > 0.05 | 1.78 | > 0.05 | 1.37 | > 0.05 | 4.29 | Negative |
| | 13-L-Hydroperoxylinoleic acid | > 0.05 | 1.12 | > 0.05 | 1.11 | > 0.05 | 0.99 | Positive |
| | 1H-Indole-3-carboxaldehyde | > 0.05 | 1.13 | > 0.05 | 1.13 | > 0.05 | 1.00 | Positive |
| | 1-Methyladenosine | > 0.05 | 0.68 | > 0.05 | 0.82 | > 0.05 | 1.21 | Positive |
| | 1-Methyl-L-histidine/3-Methyl-L-histidine | > 0.05 | 12.08 | > 0.05 | 22.77 | > 0.05 | 1.89 | Positive |
| | 2-Hydroxycinnamic acid | > 0.05 | 0.63 | > 0.05 | 0.71 | > 0.05 | 1.12 | Positive |
| | 2-Piperidinone | > 0.05 | 0.33 | > 0.05 | 0.44 | > 0.05 | 1.35 | Positive |
| | 4-Ethylbenzaldehyde | > 0.05 | 1.96 | > 0.05 | 2.25 | > 0.05 | 1.14 | Positive |
| | 4-Hydroxybenzaldehyde | > 0.05 | 0.62 | > 0.05 | 0.62 | > 0.05 | 0.99 | Positive |
| | 4-Hydroxyproline | > 0.05 | 0.29 | > 0.05 | 0.11 | > 0.05 | 0.38 | Positive |
| | 4'-Methoxychalcone | > 0.05 | 1.56 | > 0.05 | 1.24 | > 0.05 | 0.80 | Positive |
| | 6-Methylnicotinamide | > 0.05 | 2.92 | > 0.05 | 8.68 | > 0.05 | 2.98 | Positive |
| | 7-Methylguanine | > 0.05 | 0.79 | > 0.05 | 1.06 | > 0.05 | 1.34 | Positive |
| | 9-OxoODE | > 0.05 | 0.44 | > 0.05 | 0.64 | > 0.05 | 1.45 | Positive |
| | Acetylcholine | > 0.05 | 1.42 | > 0.05 | 2.22 | > 0.05 | 1.57 | Positive |
| | Adenine | > 0.05 | 0.15 | > 0.05 | 0.18 | > 0.05 | 1.18 | Positive |
| | Adenosine | > 0.05 | 0.42 | > 0.05 | 0.29 | > 0.05 | 0.70 | Positive |
| | Adipic acid | > 0.05 | 1.31 | > 0.05 | 1.67 | > 0.05 | 1.28 | Positive |
| | Alpha-N-Phenylacetyl-L-glutamine | > 0.05 | 1.01 | > 0.05 | 41.42 | > 0.05 | 40.99 | Positive |
| | Aminocaproic acid | > 0.05 | 0.75 | > 0.05 | 0.98 | > 0.05 | 1.32 | Positive |
| | Asymmetric dimethylarginine | > 0.05 | 3.22 | > 0.05 | 3.71 | > 0.05 | 1.15 | Positive |
| | Beta-Alanine | > 0.05 | 1.37 | > 0.05 | 1.01 | > 0.05 | 0.73 | Positive |
| | Betaine | > 0.05 | 0.57 | > 0.05 | 0.39 | > 0.05 | 0.68 | Positive |
| | Caffeine | > 0.05 | 0.02 | > 0.05 | 0.08 | > 0.05 | 5.29 | Positive |
| | Carnosine | > 0.05 | 5.71 | > 0.05 | 17.68 | > 0.05 | 3.10 | Positive |
| | Choline | > 0.05 | 0.43 | > 0.05 | 0.56 | > 0.05 | 1.28 | Positive |
| | Coumarin | > 0.05 | 0.64 | > 0.05 | 0.64 | > 0.05 | 1.00 | Positive |
| | Creatine | > 0.05 | 1.35 | > 0.05 | 1.12 | > 0.05 | 0.83 | Positive |
| | Creatinine | > 0.05 | 0.48 | > 0.05 | 2.30 | > 0.05 | 4.76 | Positive |
| | Cyclohexylamine | > 0.05 | 1.17 | > 0.05 | 0.74 | > 0.05 | 0.63 | Positive |
| | Deoxyinosine | > 0.05 | 3.82 | > 0.05 | 4.48 | > 0.05 | 1.17 | Positive |
| | Diaminopimelic acid | > 0.05 | 1.58 | > 0.05 | 2.82 | > 0.05 | 1.78 | Positive |
| | Ecgonine | > 0.05 | 0.82 | > 0.05 | 0.83 | > 0.05 | 1.00 | Positive |
| | Ergothioneine | > 0.05 | 0.15 | > 0.05 | 0.19 | > 0.05 | 1.24 | Positive |
| | Glycerophosphocholine | > 0.05 | 0.33 | > 0.05 | 0.40 | > 0.05 | 1.22 | Positive |
| | Guanine | > 0.05 | 1.76 | > 0.05 | 0.50 | > 0.05 | 0.28 | Positive |
| | Guanosine | > 0.05 | 1.14 | > 0.05 | 0.42 | > 0.05 | 0.37 | Positive |
| | Hexanoylcarnitine | > 0.05 | 0.77 | > 0.05 | 1.18 | > 0.05 | 1.54 | Positive |
| | Histamine | > 0.05 | 0.20 | > 0.05 | 0.68 | > 0.05 | 3.39 | Positive |
| | Hydroxyphenyllactic acid | > 0.05 | 2.10 | > 0.05 | 4.14 | > 0.05 | 1.97 | Positive |
| | Hypoxanthine | > 0.05 | 0.42 | > 0.05 | 0.69 | > 0.05 | 1.64 | Positive |
| | Indole | > 0.05 | 0.84 | > 0.05 | 1.26 | > 0.05 | 1.50 | Positive |
| | Indole-3-propionic acid | > 0.05 | 0.73 | > 0.05 | 0.84 | > 0.05 | 1.14 | Positive |
| | Inosine | > 0.05 | 0.82 | > 0.05 | 0.68 | > 0.05 | 0.83 | Positive |
| | Kanosamine | > 0.05 | 1.31 | > 0.05 | 0.51 | > 0.05 | 0.39 | Positive |
| | L-Acetylcarnitine | > 0.05 | 1.07 | > 0.05 | 2.35 | > 0.05 | 2.21 | Positive |
| | L-Alanine | > 0.05 | 1.98 | > 0.05 | 2.35 | > 0.05 | 1.19 | Positive |
| | L-Alpha-aminobutyric acid | > 0.05 | 6.42 | > 0.05 | 4.98 | > 0.05 | 0.77 | Positive |
| | L-Arginine | > 0.05 | 0.20 | > 0.05 | 0.21 | > 0.05 | 1.03 | Positive |
| | L-Carnitine | > 0.05 | 1.12 | > 0.05 | 1.10 | > 0.05 | 0.99 | Positive |
| | L-Cystine | > 0.05 | 0.19 | > 0.05 | 0.36 | > 0.05 | 1.95 | Positive |
| | Leucylproline | > 0.05 | 1.19 | > 0.05 | 2.34 | > 0.05 | 1.98 | Positive |
| | L-Glutamine | > 0.05 | 0.83 | > 0.05 | 0.65 | > 0.05 | 0.79 | Positive |
| | L-Histidine | > 0.05 | 1.01 | > 0.05 | 1.33 | > 0.05 | 1.32 | Positive |
| | L-Hypoglycin A | > 0.05 | 1.55 | > 0.05 | 0.84 | > 0.05 | 0.54 | Positive |
| | L-Isoleucine | > 0.05 | 0.24 | > 0.05 | 0.47 | > 0.05 | 1.99 | Positive |
| | L-Leucine | > 0.05 | 0.40 | > 0.05 | 0.78 | > 0.05 | 1.94 | Positive |
| | L-Lysine | > 0.05 | 0.97 | > 0.05 | 1.85 | > 0.05 | 1.91 | Positive |
| | L-Methionine | > 0.05 | 0.55 | > 0.05 | 0.64 | > 0.05 | 1.17 | Positive |
| | L-Phenylalanine | > 0.05 | 0.77 | > 0.05 | 1.07 | > 0.05 | 1.39 | Positive |
| | L-Pipecolic acid | > 0.05 | 5.36 | > 0.05 | 5.30 | > 0.05 | 0.99 | Positive |
| | L-Proline | > 0.05 | 0.72 | > 0.05 | 0.90 | > 0.05 | 1.26 | Positive |
| | L-Threonine | > 0.05 | 0.78 | > 0.05 | 1.38 | > 0.05 | 1.77 | Positive |
| | L-Tryptophan | > 0.05 | 1.09 | > 0.05 | 1.31 | > 0.05 | 1.21 | Positive |
| | L-Tyrosine | > 0.05 | 0.64 | > 0.05 | 0.75 | > 0.05 | 1.16 | Positive |
| | Methionine sulfoxide | > 0.05 | 0.24 | > 0.05 | 1.55 | > 0.05 | 6.41 | Positive |
| | N,N-Dimethylaniline | > 0.05 | 1.13 | > 0.05 | 0.91 | > 0.05 | 0.81 | Positive |
| | N1-Acetylspermine | > 0.05 | 0.39 | > 0.05 | 5.74 | > 0.05 | 14.60 | Positive |
| | N6,N6,N6-Trimethyl-L-lysine | > 0.05 | 11.79 | > 0.05 | 14.08 | > 0.05 | 1.19 | Positive |
| | N6-Acetyl-L-lysine | > 0.05 | 3.83 | > 0.05 | 17.04 | > 0.05 | 4.45 | Positive |
| | N-Acetylgalactosamine 4-sulphate | > 0.05 | 1.74 | > 0.05 | 4.09 | > 0.05 | 2.35 | Positive |
| | N-Acetyl-L-alanine | > 0.05 | 1.88 | > 0.05 | 6.39 | > 0.05 | 3.41 | Positive |
| | Niacinamide | > 0.05 | 0.10 | > 0.05 | 0.31 | > 0.05 | 3.06 | Positive |
| | Nicotinic acid | > 0.05 | 0.05 | > 0.05 | 0.07 | > 0.05 | 1.46 | Positive |
| | Octadecanamide | > 0.05 | 1.52 | > 0.05 | 1.01 | > 0.05 | 0.67 | Positive |
| | Ornithine | > 0.05 | 3.28 | > 0.05 | 5.65 | > 0.05 | 1.73 | Positive |
| | Palmitic amide | > 0.05 | 1.54 | > 0.05 | 1.06 | > 0.05 | 0.69 | Positive |
| | Phthalic acid | > 0.05 | 0.47 | > 0.05 | 0.17 | > 0.05 | 0.35 | Positive |
| | Propionylcarnitine | > 0.05 | 0.36 | > 0.05 | 1.95 | > 0.05 | 5.39 | Positive |
| | Pyroglutamic acid | > 0.05 | 0.81 | > 0.05 | 0.65 | > 0.05 | 0.79 | Positive |
| | Sphingosine | > 0.05 | 1.61 | > 0.05 | 0.42 | > 0.05 | 0.26 | Positive |
| | Taurine | > 0.05 | 0.50 | > 0.05 | 1.01 | > 0.05 | 2.02 | Positive |
| | Tranexamic acid | > 0.05 | 1.87 | > 0.05 | 2.88 | > 0.05 | 1.54 | Positive |
| | Triethanolamine | > 0.05 | 1.05 | > 0.05 | 0.94 | > 0.05 | 0.89 | Positive |
| | Uracil | > 0.05 | 0.17 | > 0.05 | 0.42 | > 0.05 | 2.44 | Positive |
| | Urea | > 0.05 | 1.56 | > 0.05 | 2.02 | > 0.05 | 1.30 | Positive |
| | Uric acid | > 0.05 | 0.84 | > 0.05 | 0.88 | > 0.05 | 1.05 | Positive |
| | Xanthine | > 0.05 | 0.35 | > 0.05 | 0.74 | > 0.05 | 2.15 | Positive |
| | Xanthosine | > 0.05 | 0.78 | > 0.05 | 5.43 | > 0.05 | 3.05 | Positive |

Table S3. Correlative analysis between adundance of different mitabolites and D-dimer, Ferritin, C-reactive protein, and different cytokines

| Spearman's correlation with significantly changed metabolites | 16-Hydroxyhexadecanoic acid | 2-Furoic acid | 3,3-Dimethylglutaric acid | 3-Methyladipic acid | Acrylic acid | Alpha-N-Phenylacetyl-L-glutamine | Ascorbic acid | Beta-D-Glucopyranuronic acid | cis-Aconitic acid | Citric acid | D-Mannose | Hydroxyphenyllactic acid | Indoxyl sulfate | Isocitric acid |
|---|---|---|---|---|---|---|---|---|---|---|---|---|---|---|
| D-dimer | 0.15 | 0.07 | 0.13 | -0.17 | -0.01 | -0.22 | 0.00 | -0.03 | 0.10 | 0.10 | 0.09 | -0.02 | -0.50 | 0.01 |
| Ferritin | 0.12 | 0.21 | 0.16 | -0.06 | -0.09 | -0.24 | -0.10 | 0.05 | 0.16 | 0.22 | -0.08 | 0.13 | -0.20 | 0.18 |
| C-reactive protein | -0.28 | -0.29 | -0.10 | 0.10 | -0.01 | 0.08 | 0.06 | 0.26 | -0.22 | -0.20 | -0.03 | 0.23 | 0.11 | -0.51 |
| G-CSF | 0.02 | 0.23 | 0.04 | 0.28 | 0.15 | -0.06 | 0.02 | 0.15 | 0.11 | 0.04 | 0.08 | 0.22 | -0.04 | -0.04 |
| IFN-a2a | 0.09 | 0.30 | 0.25 | 0.02 | 0.01 | 0.14 | 0.09 | 0.03 | 0.40 | 0.36 | -0.01 | 0.20 | 0.21 | 0.41 |
| IFN-b | -0.10 | 0.38 | -0.03 | 0.38 | 0.16 | 0.23 | 0.02 | 0.29 | 0.26 | 0.14 | 0.17 | 0.12 | 0.27 | 0.21 |
| IL-1RA | -0.21 | 0.44 | -0.01 | 0.31 | 0.10 | 0.40 | 0.31 | 0.40 | 0.40 | 0.23 | 0.26 | 0.47 | 0.53 | 0.32 |
| IL-7 | -0.35 | -0.48 | 0.06 | -0.34 | 0.19 | -0.28 | -0.25 | -0.18 | -0.34 | -0.45 | 0.03 | -0.15 | -0.10 | -0.34 |
| IL-9 | -0.22 | 0.07 | -0.27 | 0.21 | 0.34 | 0.22 | 0.23 | 0.32 | 0.04 | -0.09 | 0.39 | 0.09 | 0.18 | -0.06 |
| IP-10 | 0.21 | 0.06 | 0.51 | 0.10 | 0.21 | 0.15 | -0.02 | 0.14 | 0.40 | 0.32 | 0.21 | 0.30 | 0.11 | 0.24 |
| MCP-1 | 0.41 | 0.33 | 0.43 | 0.13 | 0.11 | 0.27 | 0.37 | 0.24 | 0.53 | 0.45 | 0.18 | 0.47 | 0.17 | 0.34 |
| MIP-1a | -0.08 | 0.13 | 0.04 | 0.19 | 0.07 | 0.12 | 0.29 | 0.31 | 0.27 | 0.27 | 0.34 | 0.42 | 0.06 | 0.15 |
| VEGF-A | -0.34 | -0.44 | -0.12 | -0.26 | 0.01 | -0.16 | -0.26 | -0.10 | -0.52 | -0.48 | -0.06 | -0.17 | -0.14 | -0.57 |
| GM-CSF | 0.05 | 0.12 | 0.14 | -0.04 | 0.03 | 0.11 | 0.21 | 0.11 | 0.17 | 0.05 | 0.04 | 0.24 | 0.09 | -0.07 |
| IFN-gamma | -0.01 | -0.07 | 0.00 | -0.11 | -0.14 | 0.16 | 0.02 | -0.05 | 0.14 | -0.03 | -0.14 | 0.20 | 0.20 | -0.06 |
| IL-10 | 0.01 | -0.01 | 0.23 | 0.07 | 0.27 | 0.08 | 0.14 | 0.17 | 0.21 | 0.18 | 0.19 | 0.40 | 0.00 | 0.00 |
| IL-12p70 | 0.07 | 0.13 | 0.11 | -0.03 | -0.12 | -0.04 | 0.18 | -0.01 | 0.09 | -0.12 | -0.15 | -0.05 | 0.06 | 0.05 |
| IL-1b | -0.18 | 0.21 | -0.06 | 0.31 | 0.14 | 0.26 | 0.12 | 0.27 | 0.22 | -0.06 | 0.26 | 0.36 | 0.43 | -0.01 |
| IL-4 | -0.17 | 0.16 | 0.08 | -0.09 | -0.24 | -0.11 | 0.26 | -0.09 | 0.11 | 0.17 | -0.23 | 0.04 | -0.09 | -0.06 |
| IL-5 | -0.14 | -0.06 | 0.14 | 0.12 | 0.09 | -0.11 | 0.22 | 0.03 | 0.01 | 0.13 | 0.23 | -0.20 | -0.03 | -0.18 |
| IL-6 | 0.05 | -0.11 | 0.22 | 0.02 | 0.22 | 0.01 | 0.23 | 0.26 | 0.16 | 0.10 | 0.27 | 0.45 | -0.09 | -0.16 |
| IL-8 | 0.39 | 0.19 | 0.24 | 0.09 | 0.05 | 0.28 | 0.26 | 0.21 | 0.27 | 0.30 | 0.15 | 0.35 | 0.19 | 0.13 |
| TNF-a | -0.08 | 0.15 | 0.16 | 0.41 | 0.25 | 0.45 | 0.42 | 0.51 | 0.43 | 0.12 | 0.48 | 0.62 | 0.45 | 0.16 |

| Spearman's correlation with significantly changed metabolites | Itaconic acid | Glycolic acid | Levulinic acid | Xanthosine | Taurine | N-Acetyl-L-alanine | L-Malic acid | Malonic acid | Methanesulfonic acid | N-Acetylglutamic acid | Orotidine | Pseudouridine | Pyroglutamic acid | Suberic acid | Succinic acid semialdehyde |
|---|---|---|---|---|---|---|---|---|---|---|---|---|---|---|---|
| D-dimer | 0.15 | 0.45 | -0.19 | -0.16 | -0.41 | -0.12 | 0.46 | 0.22 | 0.31 | -0.13 | -0.09 | -0.28 | 0.30 | -0.01 | 0.22 |
| Ferritin | 0.19 | 0.17 | -0.04 | 0.19 | -0.06 | 0.06 | -0.05 | 0.06 | 0.07 | 0.02 | 0.18 | -0.15 | -0.24 | 0.22 | 0.09 |
| C-reactive protein | -0.23 | 0.08 | 0.35 | 0.14 | -0.05 | 0.15 | -0.03 | 0.21 | 0.16 | -0.09 | 0.17 | 0.07 | 0.29 | 0.25 | -0.40 |
| G-CSF | 0.15 | 0.13 | 0.01 | 0.18 | -0.10 | 0.03 | 0.00 | 0.15 | 0.08 | 0.01 | 0.23 | -0.15 | -0.15 | 0.26 | 0.00 |
| IFN-a2a | 0.32 | 0.16 | 0.14 | 0.34 | 0.12 | 0.29 | 0.11 | 0.06 | 0.26 | 0.16 | 0.28 | -0.03 | -0.19 | 0.05 | 0.16 |
| IFN-b | 0.15 | -0.04 | 0.08 | 0.26 | -0.02 | 0.20 | -0.08 | 0.30 | 0.12 | 0.14 | 0.32 | 0.09 | -0.51 | 0.28 | -0.03 |
| IL-1RA | 0.36 | -0.06 | 0.49 | 0.59 | 0.08 | 0.58 | -0.11 | 0.21 | 0.35 | 0.52 | 0.54 | 0.43 | 0.06 | 0.15 | 0.00 |
| IL-7 | -0.16 | -0.30 | -0.01 | -0.28 | 0.09 | -0.17 | -0.14 | -0.03 | 0.20 | -0.07 | -0.24 | -0.19 | -0.03 | 0.01 | -0.33 |
| IL-9 | -0.01 | -0.20 | 0.04 | 0.14 | -0.16 | 0.13 | 0.09 | 0.30 | 0.21 | 0.30 | 0.19 | 0.27 | 0.20 | -0.10 | -0.10 |
| IP-10 | 0.39 | 0.22 | 0.08 | 0.40 | -0.35 | 0.35 | 0.11 | 0.22 | 0.55 | 0.32 | 0.39 | 0.16 | -0.07 | 0.11 | 0.26 |
| MCP-1 | 0.50 | 0.49 | -0.09 | 0.48 | -0.33 | 0.40 | 0.36 | 0.34 | 0.37 | 0.33 | 0.47 | 0.04 | 0.33 | 0.14 | 0.30 |
| MIP-1a | 0.27 | -0.09 | -0.07 | 0.45 | -0.31 | 0.42 | 0.10 | 0.50 | 0.59 | 0.49 | 0.50 | 0.19 | -0.15 | 0.33 | 0.13 |
| VEGF-A | -0.47 | -0.36 | -0.12 | -0.24 | -0.13 | -0.21 | -0.24 | -0.07 | 0.19 | -0.25 | -0.19 | -0.13 | 0.13 | -0.11 | -0.43 |
| GM-CSF | 0.27 | 0.29 | 0.10 | 0.22 | 0.08 | 0.13 | 0.24 | 0.26 | 0.05 | 0.13 | 0.27 | -0.15 | -0.17 | 0.66 | 0.03 |
| IFN-gamma | 0.07 | -0.29 | -0.01 | 0.02 | -0.13 | 0.14 | -0.05 | -0.02 | 0.27 | 0.12 | 0.09 | -0.06 | -0.13 | 0.24 | -0.08 |
| IL-10 | 0.26 | 0.18 | 0.03 | 0.30 | -0.20 | 0.28 | 0.25 | 0.31 | 0.51 | 0.23 | 0.35 | -0.10 | -0.15 | 0.52 | 0.08 |
| IL-12p70 | 0.14 | -0.04 | -0.04 | -0.09 | 0.22 | -0.04 | 0.00 | -0.02 | -0.17 | -0.06 | -0.10 | -0.08 | 0.13 | 0.25 | -0.21 |
| IL-1b | 0.32 | -0.21 | 0.21 | 0.28 | -0.08 | 0.39 | -0.14 | 0.14 | 0.37 | 0.29 | 0.30 | 0.15 | 0.03 | 0.26 | -0.01 |
| IL-4 | 0.06 | -0.01 | -0.07 | 0.16 | 0.05 | 0.01 | 0.11 | -0.04 | 0.22 | 0.05 | 0.16 | -0.21 | 0.15 | 0.25 | -0.39 |
| IL-5 | 0.03 | -0.05 | -0.26 | 0.14 | -0.16 | -0.05 | 0.00 | 0.26 | 0.29 | 0.06 | 0.14 | 0.04 | 0.27 | -0.06 | -0.08 |
| IL-6 | 0.23 | 0.27 | -0.10 | 0.33 | -0.30 | 0.30 | 0.16 | 0.46 | 0.43 | 0.32 | 0.42 | -0.08 | 0.29 | 0.37 | 0.06 |
| IL-8 | 0.28 | 0.34 | 0.09 | 0.28 | -0.19 | 0.27 | 0.22 | 0.29 | 0.23 | 0.08 | 0.25 | -0.01 | 0.22 | 0.30 | 0.33 |
| TNF-a | 0.47 | -0.06 | 0.27 | 0.65 | -0.20 | 0.70 | 0.00 | 0.48 | 0.58 | 0.69 | 0.72 | 0.54 | -0.15 | 0.62 | 0.05 |

| | 4-Dodecylbenzenesulfonic acid Na salt_NEG | 4-Hydroxyproline_NEG | Azelaic acid_NEG | D-2-Hydroxyglutaric acid_NEG | D-Glucose_NEG | Fumaric acid_NEG | Indolelactic acid_NEG | L-Lactic acid_NEG | N-Acetylisoleucine_NEG | Oxoglutaric acid_NEG | Phenyllactic acid_NEG | Pyruvic acid_NEG | Succinic acid_NEG | Uric acid_NEG | Xanthine_NEG |
|---|---|---|---|---|---|---|---|---|---|---|---|---|---|---|---|
| D-dimer | 0.06 | 0.00 | 0.20 | 0.04 | -0.01 | 0.24 | -0.20 | 0.17 | -0.05 | 0.05 | 0.04 | 0.16 | 0.08 | -0.29 | 0.09 |
| Ferritin | -0.18 | -0.01 | -0.05 | 0.29 | -0.18 | 0.24 | 0.02 | 0.24 | -0.08 | 0.03 | -0.06 | 0.06 | 0.06 | -0.23 | 0.27 |
| C-reactive protein | 0.07 | -0.41 | 0.03 | -0.08 | 0.06 | 0.02 | 0.08 | 0.23 | 0.18 | -0.25 | 0.37 | 0.06 | -0.16 | -0.51 | -0.14 |
| G-CSF | -0.10 | 0.11 | 0.10 | 0.20 | 0.13 | 0.09 | 0.17 | 0.04 | -0.13 | 0.05 | 0.21 | 0.05 | 0.08 | -0.13 | 0.15 |
| IFN-a2a | 0.05 | -0.07 | 0.28 | 0.29 | -0.01 | -0.02 | 0.34 | -0.04 | -0.19 | 0.16 | 0.06 | -0.26 | 0.01 | -0.14 | 0.28 |
| IFN-b | -0.07 | 0.49 | -0.14 | 0.33 | 0.08 | 0.05 | 0.19 | -0.03 | 0.08 | -0.05 | 0.04 | 0.22 | 0.31 | 0.06 | -0.12 |
| IL-1RA | -0.22 | 0.03 | 0.03 | 0.18 | 0.16 | -0.02 | 0.63 | -0.11 | -0.02 | -0.01 | 0.26 | -0.27 | 0.23 | -0.37 | 0.21 |
| IL-7 | 0.49 | -0.49 | 0.05 | -0.14 | 0.10 | 0.19 | -0.13 | -0.01 | 0.23 | -0.37 | -0.10 | -0.15 | -0.32 | -0.13 | -0.52 |
| IL-9 | -0.27 | 0.15 | -0.23 | -0.08 | 0.38 | 0.28 | -0.04 | 0.10 | 0.47 | 0.03 | 0.04 | 0.34 | 0.30 | 0.07 | -0.26 |
| IP-10 | 0.01 | -0.28 | 0.28 | 0.25 | 0.26 | -0.17 | 0.22 | -0.02 | 0.11 | 0.18 | 0.27 | -0.20 | -0.25 | -0.14 | 0.11 |
| MCP-1 | -0.14 | -0.03 | 0.41 | 0.30 | 0.18 | 0.09 | 0.20 | 0.09 | -0.15 | 0.26 | 0.42 | 0.14 | 0.05 | -0.26 | 0.21 |
| MIP-1a | -0.24 | 0.04 | 0.00 | 0.15 | 0.13 | 0.04 | 0.16 | -0.05 | -0.11 | 0.14 | 0.24 | -0.03 | 0.02 | -0.03 | -0.01 |
| VEGF-A | 0.50 | -0.40 | -0.12 | -0.23 | 0.09 | -0.21 | -0.18 | 0.17 | 0.20 | -0.31 | -0.03 | 0.13 | -0.31 | -0.02 | -0.34 |
| GM-CSF | 0.09 | 0.10 | 0.24 | 0.44 | -0.08 | 0.14 | 0.21 | -0.01 | -0.29 | 0.08 | 0.19 | 0.02 | 0.05 | -0.32 | -0.05 |
| IFN-gamma | 0.06 | -0.25 | -0.03 | 0.28 | -0.08 | -0.17 | 0.19 | -0.01 | -0.25 | 0.02 | 0.34 | 0.01 | -0.22 | -0.02 | -0.20 |
| IL-10 | 0.03 | -0.10 | 0.20 | 0.46 | 0.20 | 0.09 | 0.17 | 0.12 | -0.11 | -0.07 | 0.35 | -0.06 | -0.15 | -0.33 | -0.06 |
| IL-12p70 | -0.09 | 0.01 | 0.09 | 0.20 | -0.04 | 0.30 | 0.00 | 0.10 | -0.29 | -0.21 | 0.00 | 0.06 | 0.09 | 0.04 | 0.21 |
| IL-1b | 0.09 | 0.04 | -0.01 | 0.26 | 0.20 | -0.19 | 0.50 | -0.27 | -0.16 | -0.25 | 0.24 | -0.37 | 0.03 | -0.21 | -0.06 |
| IL-4 | 0.16 | -0.33 | 0.13 | 0.21 | -0.16 | 0.21 | -0.03 | 0.07 | -0.51 | 0.15 | 0.13 | 0.10 | -0.20 | -0.03 | 0.37 |
| IL-5 | 0.00 | -0.10 | -0.01 | -0.35 | 0.23 | 0.05 | -0.32 | -0.07 | 0.20 | 0.07 | -0.20 | 0.11 | -0.11 | 0.11 | -0.07 |
| IL-6 | 0.06 | -0.09 | 0.34 | 0.33 | 0.20 | 0.27 | 0.11 | 0.37 | 0.01 | 0.00 | 0.42 | 0.18 | 0.03 | -0.38 | -0.14 |
| IL-8 | -0.17 | 0.14 | 0.11 | 0.23 | 0.17 | -0.12 | 0.14 | 0.04 | -0.17 | 0.01 | 0.28 | 0.04 | 0.18 | -0.37 | 0.12 |
| TNF-a | -0.27 | 0.02 | 0.13 | 0.32 | 0.29 | 0.03 | 0.44 | -0.08 | -0.05 | -0.17 | 0.55 | -0.05 | 0.00 | -0.15 | -0.10 |

| | 12-Hydroxydodecanoic acid_NEG_B1 | 2-Hydroxy-2-methylbutyric acid_NEG_B1 | 2-Hydroxymyristic acid_NEG_B1 | 2-Hydroxyvaleric acid_NEG_B1 | 2-Phosphoglyceric acid_NEG_B1 | 3,3-Dimethylglutaric acid_NEG_B1 | 3-Hydroxycapric acid_NEG_B1 | 3-Hydroxyisovaleric acid_NEG_B1 | 3-Hydroxymethylglutaric acid_NEG_B1 | 3-Methyladipic acid_NEG_B1 | 4-Oxoproline_NEG_B1 | 6-Hydroxyhexanoic acid_NEG_B1 |
|---|---|---|---|---|---|---|---|---|---|---|---|---|
| D-dimer | 0.04 | 0.13 | 0.21 | 0.10 | -0.18 | 0.13 | 0.09 | -0.12 | -0.09 | -0.17 | 0.27 | 0.10 |
| Ferritin | 0.02 | -0.07 | 0.05 | -0.02 | 0.08 | 0.16 | -0.19 | 0.35 | 0.00 | -0.06 | 0.06 | 0.03 |
| C-reactive protein | -0.23 | 0.31 | 0.21 | -0.07 | 0.02 | -0.10 | -0.11 | 0.16 | 0.09 | 0.10 | 0.12 | 0.10 |
| G-CSF | -0.03 | -0.17 | 0.01 | -0.13 | -0.23 | 0.04 | 0.12 | 0.13 | -0.09 | 0.28 | -0.08 | -0.01 |
| IFN-a2a | 0.20 | -0.16 | 0.30 | -0.04 | 0.24 | 0.25 | 0.05 | 0.41 | 0.17 | 0.02 | 0.02 | -0.01 |
| IFN-b | -0.05 | -0.19 | -0.30 | -0.10 | -0.13 | -0.03 | -0.11 | 0.35 | 0.04 | 0.38 | -0.23 | -0.27 |
| IL-1RA | -0.21 | 0.01 | 0.17 | 0.06 | 0.33 | -0.01 | -0.21 | 0.40 | 0.59 | 0.31 | -0.03 | 0.11 |
| IL-7 | 0.12 | 0.14 | -0.06 | -0.21 | 0.05 | 0.06 | -0.14 | 0.09 | 0.05 | -0.34 | -0.11 | 0.09 |
| IL-9 | -0.32 | -0.20 | -0.24 | -0.29 | 0.07 | -0.27 | -0.25 | -0.17 | 0.21 | 0.21 | -0.17 | -0.34 |
| IP-10 | 0.20 | 0.07 | 0.25 | -0.04 | -0.04 | 0.51 | 0.20 | 0.25 | 0.24 | 0.10 | -0.14 | 0.12 |
| MCP-1 | 0.17 | 0.05 | 0.45 | 0.19 | -0.02 | 0.43 | 0.17 | 0.03 | 0.17 | 0.13 | 0.15 | -0.11 |
| MIP-1a | -0.13 | -0.06 | -0.08 | 0.03 | -0.04 | 0.04 | -0.12 | 0.07 | 0.37 | 0.19 | -0.01 | -0.05 |
| VEGF-A | -0.05 | 0.07 | 0.05 | -0.26 | -0.05 | -0.12 | 0.12 | -0.21 | -0.23 | -0.26 | -0.19 | -0.03 |
| GM-CSF | 0.15 | 0.20 | 0.15 | 0.27 | -0.17 | 0.14 | 0.15 | 0.16 | 0.06 | -0.04 | 0.17 | 0.01 |
| IFN-gamma | 0.10 | 0.18 | 0.10 | 0.05 | -0.13 | 0.00 | 0.01 | 0.09 | 0.07 | -0.11 | -0.09 | -0.12 |
| IL-10 | 0.03 | 0.08 | 0.19 | 0.01 | -0.12 | 0.23 | -0.10 | 0.30 | 0.29 | 0.07 | -0.12 | 0.23 |
| IL-12p70 | -0.07 | -0.27 | 0.12 | -0.26 | -0.18 | 0.11 | -0.26 | -0.09 | 0.00 | -0.03 | -0.11 | -0.08 |
| IL-1b | -0.06 | -0.05 | 0.21 | -0.11 | 0.08 | -0.06 | -0.09 | 0.17 | 0.40 | 0.31 | -0.05 | 0.25 |
| IL-4 | -0.27 | -0.17 | 0.19 | -0.14 | -0.29 | 0.08 | 0.03 | 0.02 | 0.05 | -0.09 | -0.18 | -0.18 |
| IL-5 | 0.03 | -0.19 | 0.08 | -0.31 | -0.12 | 0.14 | 0.16 | -0.36 | -0.03 | 0.12 | 0.08 | -0.39 |
| IL-6 | -0.12 | 0.11 | 0.34 | -0.04 | -0.15 | 0.22 | -0.06 | -0.02 | 0.26 | 0.02 | 0.07 | 0.19 |
| IL-8 | 0.15 | 0.16 | 0.25 | 0.20 | 0.02 | 0.24 | 0.05 | -0.03 | -0.08 | 0.09 | 0.29 | 0.05 |
| TNF-a | 0.00 | 0.23 | 0.13 | 0.17 | -0.10 | 0.16 | -0.29 | 0.31 | 0.67 | 0.41 | -0.25 | 0.20 |

| | Adipic acid_NEG_B1 | Alpha-Lactose_NEG_B1 | Citraconic acid_NEG_B1 | Glucaric acid_NEG_B1 | Inosine_NEG_B1 | L-Aspartic acid_NEG_B1 | L-Glyceric acid_NEG_B1 | Methylsuccinic acid_NEG_B1 | Oxalic acid_NEG_B1 | Ribonolactone_NEG_B1 | Saccharin_NEG_B1 | Tartaric acid_NEG_B1 | 1-Methyl-L-histidine/3-Methyl-L-histidine_POS_B1 |
|---|---|---|---|---|---|---|---|---|---|---|---|---|---|
| D-dimer | -0.02 | 0.01 | -0.03 | 0.11 | 0.31 | 0.02 | 0.30 | -0.02 | 0.27 | 0.04 | 0.41 | -0.13 | 0.08 |
| Ferritin | 0.18 | -0.09 | -0.23 | -0.02 | 0.24 | -0.05 | 0.19 | -0.16 | 0.09 | 0.27 | 0.39 | 0.19 | 0.20 |
| C-reactive protein | 0.17 | 0.00 | -0.24 | 0.11 | -0.15 | -0.07 | -0.28 | 0.05 | -0.35 | 0.17 | 0.19 | 0.02 | -0.05 |
| G-CSF | 0.17 | 0.12 | -0.19 | -0.05 | 0.09 | -0.06 | 0.16 | 0.18 | 0.13 | 0.34 | 0.43 | 0.24 | 0.17 |
| IFN-a2a | -0.15 | -0.14 | -0.29 | 0.01 | 0.18 | -0.08 | 0.05 | -0.01 | 0.12 | 0.00 | 0.07 | 0.16 | 0.36 |
| IFN-b | 0.15 | 0.26 | 0.03 | 0.10 | -0.35 | -0.30 | -0.01 | 0.06 | 0.30 | 0.37 | 0.50 | 0.44 | 0.10 |
| IL-1RA | 0.21 | 0.12 | -0.20 | 0.11 | 0.06 | -0.27 | -0.14 | 0.15 | 0.16 | 0.03 | 0.07 | 0.35 | 0.67 |
| IL-7 | -0.10 | -0.14 | -0.05 | -0.35 | -0.19 | -0.06 | -0.06 | 0.14 | -0.23 | 0.03 | 0.17 | -0.35 | -0.32 |
| IL-9 | 0.29 | 0.39 | 0.08 | -0.07 | -0.26 | -0.31 | 0.03 | 0.30 | 0.26 | 0.27 | 0.35 | 0.28 | 0.07 |
| IP-10 | 0.06 | 0.01 | -0.50 | 0.11 | -0.04 | -0.21 | 0.07 | 0.26 | -0.04 | 0.20 | 0.17 | 0.23 | 0.21 |
| MCP-1 | 0.11 | 0.06 | -0.41 | 0.19 | 0.02 | 0.09 | 0.03 | 0.15 | 0.09 | 0.28 | 0.23 | 0.44 | 0.32 |
| MIP-1a | 0.23 | 0.19 | -0.46 | -0.10 | -0.01 | -0.32 | 0.14 | 0.09 | 0.23 | -0.04 | 0.41 | 0.41 | 0.37 |
| VEGF-A | -0.05 | 0.23 | -0.20 | -0.07 | -0.21 | 0.18 | -0.19 | 0.07 | -0.34 | 0.12 | -0.16 | -0.38 | -0.44 |
| GM-CSF | 0.27 | -0.11 | -0.30 | 0.13 | -0.09 | 0.02 | -0.04 | -0.06 | -0.09 | 0.23 | 0.35 | 0.09 | 0.11 |
| IFN-gamma | -0.26 | -0.09 | -0.34 | -0.23 | -0.37 | -0.12 | -0.17 | -0.05 | -0.09 | 0.29 | -0.03 | 0.07 | -0.17 |
| IL-10 | 0.16 | 0.07 | -0.51 | 0.09 | 0.09 | -0.21 | -0.04 | 0.25 | -0.05 | 0.18 | 0.54 | 0.37 | 0.22 |
| IL-12p70 | -0.04 | -0.09 | 0.18 | -0.05 | 0.28 | -0.02 | 0.21 | 0.01 | 0.03 | 0.13 | 0.08 | 0.17 | 0.10 |
| IL-1b | 0.09 | 0.17 | -0.20 | 0.05 | -0.01 | -0.31 | -0.23 | 0.20 | -0.10 | 0.00 | 0.34 | 0.16 | 0.43 |
| IL-4 | -0.14 | 0.02 | -0.37 | -0.30 | 0.03 | -0.03 | -0.06 | -0.16 | -0.24 | 0.03 | -0.15 | 0.01 | 0.15 |
| IL-5 | 0.22 | 0.17 | -0.11 | -0.11 | -0.28 | -0.15 | 0.09 | 0.13 | -0.23 | 0.01 | 0.12 | 0.03 | -0.08 |
| IL-6 | 0.26 | 0.15 | -0.50 | -0.01 | 0.08 | -0.19 | 0.05 | 0.19 | -0.17 | 0.15 | 0.49 | 0.37 | 0.26 |
| IL-8 | 0.11 | 0.03 | -0.17 | 0.41 | 0.26 | 0.02 | -0.09 | 0.15 | 0.11 | 0.29 | 0.33 | 0.34 | 0.04 |
| TNF-a | 0.38 | 0.14 | -0.29 | 0.10 | -0.28 | -0.25 | -0.06 | 0.29 | -0.12 | 0.18 | 0.23 | 0.61 | 0.53 |

Unchanged metabolites

| | 2-Hydroxycinnamic acid_POS_B1 | 3-(4-Methoxyphenyl)-2-propenal_POS_B1 | 3,4-Dihydroxyhydrocinnamic acid_POS_B1 | 3-Methylhistidine_POS_B1 | 4-Ethylbenzaldehyde_POS_B1 | 6-Methylnicotinamide_POS_B1 | Adenosine_POS_B1 | Citrulline_POS_B1 | Dibutyl phthalate_POS_B1 | L-Glutamic acid_POS_B1 | L-Hypoglycin A_POS_B1 | L-Serine_POS_B1 |
|---|---|---|---|---|---|---|---|---|---|---|---|---|
| D-dimer | 0.18 | 0.11 | -0.17 | 0.06 | 0.17 | -0.39 | 0.05 | 0.21 | 0.16 | 0.21 | -0.16 | 0.30 |
| Ferritin | -0.03 | 0.02 | 0.23 | 0.11 | 0.00 | 0.18 | 0.44 | 0.02 | 0.04 | 0.01 | -0.07 | 0.14 |
| C-reactive protein | 0.05 | 0.17 | 0.07 | -0.43 | -0.03 | 0.04 | 0.36 | -0.04 | 0.07 | 0.10 | -0.08 | -0.50 |
| G-CSF | 0.05 | 0.05 | 0.13 | 0.08 | 0.03 | 0.03 | 0.24 | -0.10 | -0.04 | -0.18 | -0.11 | -0.02 |
| IFN-a2a | -0.14 | 0.01 | 0.20 | 0.47 | -0.16 | 0.37 | 0.01 | 0.07 | -0.02 | 0.08 | -0.35 | -0.07 |
| IFN-b | -0.03 | 0.00 | 0.06 | 0.04 | 0.14 | 0.14 | 0.10 | 0.05 | -0.21 | -0.39 | 0.10 | -0.04 |
| IL-1RA | 0.01 | 0.01 | 0.35 | 0.32 | -0.07 | 0.70 | 0.13 | 0.06 | -0.35 | 0.02 | -0.03 | -0.38 |
| IL-7 | 0.12 | 0.35 | 0.22 | -0.23 | 0.18 | -0.07 | 0.01 | 0.17 | 0.06 | 0.39 | 0.21 | -0.09 |
| IL-9 | 0.19 | -0.11 | 0.02 | -0.26 | 0.00 | 0.06 | 0.20 | 0.09 | 0.16 | 0.08 | 0.36 | -0.25 |
| IP-10 | -0.19 | -0.16 | -0.04 | 0.42 | -0.29 | 0.12 | -0.09 | 0.12 | -0.23 | 0.21 | -0.19 | -0.01 |
| MCP-1 | -0.19 | -0.15 | -0.02 | 0.43 | -0.21 | 0.17 | -0.14 | -0.02 | 0.16 | -0.05 | -0.21 | -0.14 |
| MIP-1a | 0.03 | -0.15 | 0.11 | 0.21 | 0.06 | 0.41 | 0.12 | 0.04 | -0.20 | 0.00 | 0.11 | 0.07 |
| VEGF-A | 0.27 | 0.30 | -0.22 | -0.27 | -0.14 | -0.35 | 0.04 | -0.16 | -0.05 | 0.39 | 0.24 | -0.09 |
| GM-CSF | -0.02 | 0.15 | -0.07 | 0.05 | 0.31 | 0.11 | 0.10 | 0.06 | 0.10 | -0.13 | -0.37 | 0.12 |
| IFN-gamma | 0.07 | 0.13 | 0.16 | 0.04 | -0.25 | 0.17 | -0.06 | 0.00 | 0.01 | -0.15 | -0.29 | -0.15 |
| IL-10 | -0.08 | -0.01 | 0.08 | 0.17 | 0.09 | 0.20 | 0.28 | 0.11 | 0.01 | 0.12 | -0.21 | -0.21 |
| IL-12p70 | -0.32 | -0.13 | 0.27 | 0.09 | 0.02 | -0.18 | 0.05 | -0.13 | 0.17 | -0.07 | -0.14 | -0.09 |
| IL-1b | -0.01 | -0.06 | 0.35 | 0.36 | -0.12 | 0.34 | 0.10 | 0.03 | -0.22 | 0.12 | -0.12 | -0.40 |
| IL-4 | -0.19 | 0.15 | 0.23 | 0.03 | 0.13 | -0.01 | 0.13 | -0.44 | 0.15 | 0.16 | -0.20 | 0.03 |
| IL-5 | -0.05 | -0.34 | -0.18 | -0.02 | -0.20 | -0.12 | -0.01 | -0.41 | -0.04 | 0.26 | 0.20 | 0.17 |
| IL-6 | 0.00 | 0.00 | 0.01 | -0.03 | 0.12 | 0.15 | 0.31 | -0.13 | 0.09 | 0.19 | -0.14 | -0.27 |
| IL-8 | -0.15 | -0.21 | -0.15 | 0.27 | -0.29 | -0.04 | -0.04 | 0.26 | 0.15 | -0.24 | -0.40 | -0.22 |
| TNF-a | -0.21 | -0.15 | 0.24 | 0.22 | -0.06 | 0.60 | 0.01 | 0.08 | -0.24 | -0.07 | 0.05 | -0.36 |

| | N-Acetylarylamine_POS_B1 | Oleamide_POS_B1 | o-Tyrosine_POS_B1 | Pipecolic acid_POS_B1 | Proline betaine_POS_B1 | Symmetric dimethylarginine_POS_B1 | Triethanolamine_POS_B1 | Trigonelline_POS_B1 | 1-Methy-L-lhistidine/3-Methyl-L-histidine_POS | 4-Hydroxybenzaldehyde_POS | 6-Pentyl-2H-pyran-2-one_POS | Betaine_POS | Choline_POS |
|---|---|---|---|---|---|---|---|---|---|---|---|---|---|
| D-dimer | -0.29 | 0.14 | -0.03 | 0.13 | 0.27 | -0.18 | -0.03 | -0.08 | 0.09 | 0.21 | 0.02 | -0.12 | -0.27 |
| Ferritin | 0.14 | -0.07 | -0.37 | 0.16 | 0.15 | 0.06 | -0.11 | 0.25 | 0.10 | 0.25 | -0.05 | 0.24 | 0.13 |
| C-reactive protein | -0.07 | 0.15 | 0.21 | -0.09 | -0.18 | -0.01 | -0.10 | -0.21 | -0.07 | -0.10 | -0.20 | 0.22 | 0.26 |
| G-CSF | 0.23 | 0.17 | 0.08 | 0.10 | 0.08 | 0.09 | -0.17 | 0.15 | 0.17 | 0.08 | -0.37 | -0.10 | 0.02 |
| IFN-a2a | 0.11 | -0.26 | -0.17 | 0.02 | 0.33 | 0.09 | 0.09 | 0.42 | 0.31 | 0.06 | -0.27 | -0.01 | 0.13 |
| IFN-b | 0.20 | 0.42 | -0.14 | -0.11 | -0.08 | 0.35 | -0.40 | 0.26 | 0.18 | 0.12 | -0.09 | 0.20 | 0.30 |
| IL-1RA | 0.26 | -0.18 | -0.03 | 0.19 | 0.11 | 0.46 | -0.19 | 0.29 | 0.46 | 0.30 | -0.42 | 0.06 | 0.52 |
| IL-7 | 0.09 | 0.34 | 0.19 | -0.28 | -0.16 | -0.43 | 0.17 | -0.23 | -0.23 | 0.16 | 0.29 | 0.04 | -0.13 |
| IL-9 | 0.05 | 0.48 | 0.16 | 0.04 | -0.14 | 0.22 | -0.31 | -0.09 | 0.02 | 0.42 | 0.14 | -0.02 | 0.38 |
| IP-10 | -0.08 | 0.09 | 0.31 | -0.26 | 0.28 | 0.25 | 0.10 | 0.17 | 0.38 | 0.00 | -0.33 | 0.30 | 0.10 |
| MCP-1 | -0.06 | 0.21 | -0.05 | 0.02 | 0.36 | 0.25 | 0.01 | 0.43 | 0.50 | 0.00 | -0.38 | -0.01 | 0.15 |
| MIP-1a | 0.25 | 0.43 | 0.10 | 0.11 | 0.14 | 0.38 | -0.20 | 0.32 | 0.22 | 0.38 | -0.31 | 0.20 | 0.32 |
| VEGF-A | -0.36 | 0.15 | 0.39 | -0.40 | -0.15 | -0.32 | 0.19 | -0.25 | -0.35 | -0.14 | -0.02 | 0.17 | -0.05 |
| GM-CSF | -0.03 | 0.32 | -0.18 | 0.09 | -0.03 | -0.02 | -0.06 | 0.32 | 0.23 | 0.06 | -0.18 | 0.05 | 0.06 |
| IFN-gamma | 0.09 | 0.34 | 0.19 | -0.24 | -0.02 | -0.03 | 0.07 | 0.22 | -0.12 | -0.13 | -0.18 | 0.21 | 0.00 |
| IL-10 | 0.14 | 0.31 | 0.12 | 0.02 | 0.03 | 0.06 | -0.08 | 0.30 | 0.19 | 0.25 | -0.25 | 0.16 | 0.15 |
| IL-12p70 | 0.33 | -0.13 | 0.00 | -0.06 | 0.32 | -0.02 | 0.10 | 0.16 | 0.01 | -0.19 | -0.09 | -0.33 | -0.30 |
| IL-1b | 0.23 | 0.11 | 0.03 | -0.08 | 0.14 | 0.22 | -0.03 | 0.42 | 0.31 | 0.12 | -0.33 | -0.08 | 0.21 |
| IL-4 | -0.06 | -0.14 | 0.27 | -0.06 | 0.24 | -0.11 | 0.34 | 0.18 | -0.07 | 0.17 | -0.43 | 0.24 | -0.04 |
| IL-5 | 0.05 | 0.29 | 0.15 | -0.26 | 0.16 | 0.12 | 0.11 | 0.03 | -0.05 | 0.12 | -0.19 | 0.48 | 0.13 |
| IL-6 | 0.06 | 0.34 | 0.07 | -0.08 | 0.04 | 0.03 | -0.09 | 0.25 | 0.13 | 0.26 | -0.26 | 0.16 | 0.24 |
| IL-8 | -0.01 | 0.28 | -0.16 | 0.10 | 0.18 | 0.12 | -0.07 | 0.37 | 0.32 | -0.21 | -0.14 | -0.30 | 0.01 |
| TNF-a | 0.18 | 0.31 | 0.16 | -0.08 | 0.04 | 0.63 | -0.29 | 0.41 | 0.46 | 0.07 | -0.46 | 0.33 | 0.48 |

| | Creatine_POS | Creatinine_POS | Ecgonine_POS | Glutarylcarnitine_POS | Hypoxanthine_POS | Indole-3-propionic acid_POS | L-Acetylcarnitine_POS | L-Alanine_POS | L-Alpha-aminobutyric acid_POS | L-Arginine_POS | L-Carnitine_POS | L-Cystine_POS |
|---|---|---|---|---|---|---|---|---|---|---|---|---|
| D-dimer | -0.15 | -0.09 | 0.15 | -0.06 | 0.10 | 0.09 | -0.06 | -0.08 | -0.07 | -0.12 | 0.16 | 0.06 |
| Ferritin | 0.25 | 0.19 | 0.14 | 0.03 | 0.19 | 0.03 | 0.30 | 0.16 | 0.21 | -0.17 | 0.00 | -0.03 |
| C-reactive protein | -0.04 | 0.18 | -0.30 | 0.31 | -0.22 | -0.05 | -0.23 | -0.04 | 0.04 | -0.11 | -0.14 | -0.35 |
| G-CSF | 0.07 | 0.26 | -0.04 | 0.14 | -0.17 | -0.07 | 0.05 | 0.03 | 0.09 | -0.36 | -0.16 | -0.08 |
| IFN-a2a | -0.20 | 0.43 | -0.15 | 0.11 | 0.04 | 0.04 | 0.39 | 0.44 | 0.16 | -0.19 | -0.19 | 0.01 |
| IFN-b | 0.38 | 0.29 | 0.14 | 0.21 | -0.27 | -0.29 | 0.14 | 0.00 | -0.03 | -0.31 | -0.03 | -0.02 |
| IL-1RA | -0.02 | 0.65 | -0.16 | 0.47 | -0.14 | 0.10 | 0.39 | 0.46 | 0.40 | -0.10 | -0.05 | 0.10 |
| IL-7 | -0.26 | -0.12 | -0.39 | -0.15 | -0.12 | 0.14 | -0.43 | -0.10 | -0.01 | -0.04 | -0.30 | -0.11 |
| IL-9 | 0.14 | 0.12 | 0.02 | 0.17 | -0.12 | -0.10 | -0.30 | -0.08 | 0.00 | -0.07 | 0.18 | -0.18 |
| IP-10 | -0.41 | 0.46 | -0.28 | 0.12 | -0.15 | -0.04 | 0.00 | 0.34 | 0.24 | -0.33 | -0.19 | -0.02 |
| MCP-1 | -0.35 | 0.54 | -0.26 | 0.23 | -0.15 | -0.13 | 0.11 | 0.38 | 0.03 | -0.39 | -0.19 | -0.27 |
| MIP-1a | -0.24 | 0.46 | -0.27 | 0.29 | -0.25 | -0.26 | -0.04 | 0.10 | 0.48 | -0.50 | -0.25 | -0.08 |
| VEGF-A | -0.23 | -0.22 | -0.20 | -0.13 | -0.11 | -0.05 | -0.54 | -0.19 | -0.15 | -0.04 | 0.08 | -0.22 |
| GM-CSF | 0.23 | 0.24 | -0.22 | 0.09 | -0.31 | 0.18 | 0.04 | 0.19 | -0.18 | -0.19 | -0.34 | 0.03 |
| IFN-gamma | -0.18 | 0.17 | -0.53 | -0.01 | -0.43 | -0.11 | -0.37 | 0.27 | 0.20 | -0.32 | -0.61 | -0.31 |
| IL-10 | -0.04 | 0.38 | -0.37 | 0.19 | -0.23 | -0.05 | -0.07 | 0.33 | 0.26 | -0.41 | -0.43 | -0.15 |
| IL-12p70 | 0.14 | 0.00 | 0.04 | -0.14 | 0.07 | -0.23 | 0.28 | -0.01 | -0.22 | -0.23 | -0.22 | -0.19 |
| IL-1b | 0.07 | 0.46 | -0.35 | 0.28 | -0.34 | -0.07 | 0.22 | 0.25 | 0.14 | -0.28 | -0.36 | 0.08 |
| IL-4 | -0.19 | 0.06 | 0.02 | -0.13 | -0.18 | -0.18 | 0.17 | 0.24 | 0.12 | -0.46 | -0.10 | -0.26 |
| IL-5 | -0.20 | -0.02 | 0.08 | -0.11 | -0.17 | -0.34 | -0.09 | -0.01 | -0.03 | -0.28 | 0.12 | -0.23 |
| IL-6 | -0.15 | 0.35 | -0.35 | 0.30 | -0.21 | -0.11 | -0.20 | 0.29 | 0.06 | -0.46 | -0.25 | -0.26 |
| IL-8 | -0.11 | 0.36 | -0.30 | 0.22 | 0.03 | -0.13 | 0.12 | 0.07 | -0.13 | -0.14 | -0.20 | -0.04 |
| TNF-a | -0.05 | 0.69 | -0.40 | 0.46 | -0.54 | -0.27 | 0.02 | 0.39 | 0.42 | -0.40 | -0.39 | -0.19 |

| | L-Glutamine_POS | L-Histidine_POS | L-Isoleucine_POS | L-Leucine_POS | L-Methionine_POS | L-Phenylalanine_POS | L-Proline_POS | L-Tryptophan_1_POS | L-Tyrosine_POS | Methacholine_POS | Methylimidazoleacetic acid_POS | N,N-Dimethylaniline_POS | N6,N6,N6-Trimethyl-L-lysine_POS | N-Acetylornithine_POS | Octadecanamide_POS | Ornithine_POS | Palmitic amide_POS |
|---|---|---|---|---|---|---|---|---|---|---|---|---|---|---|---|---|---|
| D-dimer | 0.01 | 0.44 | -0.11 | -0.06 | -0.10 | 0.07 | -0.18 | 0.19 | 0.25 | -0.22 | 0.04 | -0.43 | -0.17 | 0.06 | 0.10 | 0.12 | 0.12 |
| Ferritin | 0.06 | 0.44 | 0.14 | 0.13 | 0.13 | 0.02 | -0.23 | 0.17 | 0.14 | 0.02 | 0.15 | 0.09 | 0.20 | -0.04 | 0.01 | 0.07 | 0.11 |
| C-reactive protein | -0.34 | 0.07 | 0.28 | 0.36 | 0.09 | 0.62 | -0.06 | -0.04 | 0.20 | -0.15 | 0.14 | -0.01 | 0.24 | -0.41 | 0.13 | 0.65 | -0.02 |
| G-CSF | -0.07 | 0.42 | -0.03 | 0.07 | -0.01 | 0.15 | -0.28 | 0.06 | 0.12 | 0.03 | 0.34 | 0.10 | 0.18 | 0.01 | -0.17 | 0.05 | -0.07 |
| IFN-a2a | 0.21 | 0.47 | 0.05 | 0.09 | -0.05 | 0.04 | -0.35 | 0.03 | -0.05 | 0.14 | 0.30 | -0.04 | -0.03 | 0.17 | -0.04 | -0.10 | 0.01 |
| IFN-b | 0.27 | 0.26 | -0.30 | -0.24 | 0.09 | -0.13 | 0.08 | -0.20 | -0.02 | 0.24 | 0.37 | 0.20 | 0.14 | 0.05 | -0.21 | -0.15 | -0.26 |
| IL-1RA | 0.18 | 0.32 | 0.32 | 0.34 | 0.26 | 0.31 | -0.04 | 0.03 | 0.14 | 0.23 | 0.50 | -0.06 | 0.41 | 0.00 | -0.16 | 0.18 | -0.22 |
| IL-7 | -0.12 | -0.12 | 0.13 | 0.11 | -0.05 | 0.06 | 0.06 | 0.06 | 0.17 | -0.22 | -0.27 | -0.07 | 0.01 | -0.17 | 0.12 | -0.01 | 0.06 |
| IL-9 | 0.19 | -0.22 | -0.06 | -0.10 | 0.11 | 0.11 | 0.26 | -0.09 | 0.21 | 0.16 | 0.25 | -0.14 | 0.22 | -0.12 | -0.14 | 0.28 | -0.14 |
| IP-10 | -0.03 | 0.23 | -0.12 | -0.04 | -0.38 | 0.11 | -0.47 | -0.10 | -0.16 | 0.29 | 0.28 | 0.29 | 0.30 | 0.14 | 0.18 | 0.19 | 0.15 |
| MCP-1 | 0.10 | 0.50 | -0.15 | -0.06 | -0.26 | 0.04 | -0.45 | -0.16 | -0.13 | 0.30 | 0.56 | 0.10 | 0.12 | 0.07 | 0.10 | 0.22 | -0.11 |
| MIP-1a | 0.16 | 0.27 | -0.15 | -0.15 | -0.02 | -0.04 | -0.01 | -0.15 | 0.14 | 0.19 | 0.49 | 0.15 | 0.40 | 0.00 | -0.18 | 0.06 | -0.15 |
| VEGF-A | -0.32 | -0.30 | -0.08 | -0.06 | -0.22 | 0.21 | 0.03 | -0.09 | 0.18 | -0.07 | -0.27 | 0.02 | 0.04 | 0.09 | 0.33 | 0.23 | 0.11 |
| GM-CSF | 0.01 | 0.58 | 0.19 | 0.27 | 0.10 | 0.17 | -0.23 | 0.10 | 0.13 | -0.35 | 0.31 | -0.06 | -0.02 | -0.11 | -0.10 | 0.20 | -0.11 |
| IFN-gamma | -0.02 | 0.01 | -0.05 | 0.01 | 0.00 | 0.09 | -0.05 | -0.18 | 0.11 | -0.19 | 0.20 | 0.09 | 0.03 | 0.02 | -0.19 | 0.24 | -0.22 |
| IL-10 | 0.05 | 0.44 | 0.12 | 0.16 | -0.02 | 0.17 | -0.23 | -0.01 | 0.20 | -0.19 | 0.39 | 0.09 | 0.23 | -0.18 | -0.03 | 0.19 | 0.09 |
| IL-12p70 | -0.04 | -0.01 | -0.14 | -0.13 | 0.00 | -0.07 | 0.01 | -0.17 | -0.16 | -0.20 | 0.21 | -0.31 | -0.15 | 0.04 | -0.27 | -0.21 | -0.22 |
| IL-1b | 0.05 | 0.36 | 0.25 | 0.21 | 0.08 | 0.17 | -0.14 | -0.05 | 0.19 | -0.17 | 0.54 | -0.14 | 0.20 | 0.06 | 0.04 | -0.11 | 0.09 |
| IL-4 | -0.01 | 0.09 | 0.00 | 0.10 | -0.19 | 0.37 | -0.09 | -0.20 | -0.06 | -0.10 | 0.13 | 0.09 | -0.13 | 0.17 | -0.21 | 0.29 | -0.10 |
| IL-5 | 0.04 | -0.17 | -0.22 | -0.27 | -0.27 | 0.03 | 0.01 | -0.36 | -0.17 | 0.25 | 0.07 | 0.39 | 0.11 | -0.02 | -0.09 | 0.24 | -0.04 |
| IL-6 | -0.15 | 0.37 | 0.15 | 0.17 | -0.09 | 0.20 | -0.15 | -0.10 | 0.25 | -0.12 | 0.45 | -0.04 | 0.28 | -0.15 | 0.19 | 0.41 | 0.08 |
| IL-8 | 0.16 | 0.57 | -0.08 | -0.03 | 0.00 | 0.08 | -0.33 | 0.00 | -0.02 | -0.05 | 0.50 | -0.14 | 0.04 | -0.17 | 0.08 | -0.06 | -0.08 |
| TNF-a | -0.08 | 0.14 | 0.00 | -0.01 | -0.05 | 0.07 | -0.02 | -0.35 | -0.03 | 0.07 | 0.69 | 0.27 | 0.63 | -0.05 | -0.01 | 0.23 | -0.19 |

| Penbutolol_POS | Tranexamic Acid_POS | Urea_POS |
|---|---|---|
| 0.22 | 0.26 | -0.33 |
| -0.10 | 0.14 | -0.14 |
| 0.02 | 0.12 | 0.08 |
| -0.06 | 0.03 | -0.01 |
| -0.10 | 0.18 | 0.01 |
| -0.20 | -0.16 | 0.15 |
| -0.33 | 0.06 | 0.37 |
| 0.05 | -0.05 | -0.07 |
| -0.26 | -0.25 | 0.24 |
| -0.12 | 0.32 | 0.26 |
| 0.00 | 0.35 | 0.13 |
| 0.01 | -0.03 | 0.13 |
| 0.08 | 0.08 | 0.01 |
| -0.16 | 0.17 | -0.06 |
| -0.09 | -0.13 | -0.03 |
| -0.13 | 0.01 | -0.05 |
| 0.21 | 0.04 | -0.31 |
| -0.15 | 0.09 | 0.12 |
| -0.03 | 0.00 | -0.34 |
| 0.11 | 0.06 | 0.00 |
| -0.02 | 0.16 | -0.02 |
| 0.26 | 0.34 | 0.03 |
| -0.23 | 0.04 | 0.38 |
